# SlALKBH9B is involved in drought-induced flower drop by regulating ethylene production

**DOI:** 10.1093/hr/uhaf173

**Published:** 2025-07-07

**Authors:** Yue Cai, Lina Cheng, Xianfeng Liu, Ruizhen Li, Yang Liu, Siqi Ge, Sai Wang, Jing Liu, Changhua Tan, Sida Meng, Mingfang Qi, Cai-Zhong Jiang, Tianlai Li, Tao Xu

**Affiliations:** College of Horticulture, Shenyang Agricultural University, No. 120, Dongling Road, Shenyang, Liaoning 110866, China; Key Laboratory of Protected Horticulture of Ministry of Education, No. 120, Dongling Road, Shenyang 110866, China; Modern Protected Horticulture Engineering & Technology Center, Shenyang Agricultural University, No. 120, Dongling Road, Shenyang 110866, China; College of Horticulture, Shenyang Agricultural University, No. 120, Dongling Road, Shenyang, Liaoning 110866, China; Key Laboratory of Protected Horticulture of Ministry of Education, No. 120, Dongling Road, Shenyang 110866, China; Modern Protected Horticulture Engineering & Technology Center, Shenyang Agricultural University, No. 120, Dongling Road, Shenyang 110866, China; College of Horticulture, Shenyang Agricultural University, No. 120, Dongling Road, Shenyang, Liaoning 110866, China; Key Laboratory of Protected Horticulture of Ministry of Education, No. 120, Dongling Road, Shenyang 110866, China; Modern Protected Horticulture Engineering & Technology Center, Shenyang Agricultural University, No. 120, Dongling Road, Shenyang 110866, China; College of Horticulture, Shenyang Agricultural University, No. 120, Dongling Road, Shenyang, Liaoning 110866, China; Key Laboratory of Protected Horticulture of Ministry of Education, No. 120, Dongling Road, Shenyang 110866, China; Modern Protected Horticulture Engineering & Technology Center, Shenyang Agricultural University, No. 120, Dongling Road, Shenyang 110866, China; College of Horticulture, Shenyang Agricultural University, No. 120, Dongling Road, Shenyang, Liaoning 110866, China; Key Laboratory of Protected Horticulture of Ministry of Education, No. 120, Dongling Road, Shenyang 110866, China; Modern Protected Horticulture Engineering & Technology Center, Shenyang Agricultural University, No. 120, Dongling Road, Shenyang 110866, China; College of Horticulture, Shenyang Agricultural University, No. 120, Dongling Road, Shenyang, Liaoning 110866, China; Key Laboratory of Protected Horticulture of Ministry of Education, No. 120, Dongling Road, Shenyang 110866, China; Modern Protected Horticulture Engineering & Technology Center, Shenyang Agricultural University, No. 120, Dongling Road, Shenyang 110866, China; College of Horticulture, Shenyang Agricultural University, No. 120, Dongling Road, Shenyang, Liaoning 110866, China; Key Laboratory of Protected Horticulture of Ministry of Education, No. 120, Dongling Road, Shenyang 110866, China; Modern Protected Horticulture Engineering & Technology Center, Shenyang Agricultural University, No. 120, Dongling Road, Shenyang 110866, China; College of Horticulture, Shenyang Agricultural University, No. 120, Dongling Road, Shenyang, Liaoning 110866, China; Key Laboratory of Protected Horticulture of Ministry of Education, No. 120, Dongling Road, Shenyang 110866, China; Modern Protected Horticulture Engineering & Technology Center, Shenyang Agricultural University, No. 120, Dongling Road, Shenyang 110866, China; College of Horticulture, Shenyang Agricultural University, No. 120, Dongling Road, Shenyang, Liaoning 110866, China; Key Laboratory of Protected Horticulture of Ministry of Education, No. 120, Dongling Road, Shenyang 110866, China; Modern Protected Horticulture Engineering & Technology Center, Shenyang Agricultural University, No. 120, Dongling Road, Shenyang 110866, China; College of Horticulture, Shenyang Agricultural University, No. 120, Dongling Road, Shenyang, Liaoning 110866, China; Key Laboratory of Protected Horticulture of Ministry of Education, No. 120, Dongling Road, Shenyang 110866, China; Modern Protected Horticulture Engineering & Technology Center, Shenyang Agricultural University, No. 120, Dongling Road, Shenyang 110866, China; College of Horticulture, Shenyang Agricultural University, No. 120, Dongling Road, Shenyang, Liaoning 110866, China; Key Laboratory of Protected Horticulture of Ministry of Education, No. 120, Dongling Road, Shenyang 110866, China; Modern Protected Horticulture Engineering & Technology Center, Shenyang Agricultural University, No. 120, Dongling Road, Shenyang 110866, China; Crops Pathology and Genetic Research Unit, United States Department of Agriculture, Agricultural Research Service, Davis, 1 Shields Ave, Davis, California 95616, USA; Department of Plant Sciences, University of California at Davis, 1 Shields Ave, Davis, California 95616, USA; College of Horticulture, Shenyang Agricultural University, No. 120, Dongling Road, Shenyang, Liaoning 110866, China; Key Laboratory of Protected Horticulture of Ministry of Education, No. 120, Dongling Road, Shenyang 110866, China; Modern Protected Horticulture Engineering & Technology Center, Shenyang Agricultural University, No. 120, Dongling Road, Shenyang 110866, China; College of Horticulture, Shenyang Agricultural University, No. 120, Dongling Road, Shenyang, Liaoning 110866, China; Key Laboratory of Protected Horticulture of Ministry of Education, No. 120, Dongling Road, Shenyang 110866, China; Modern Protected Horticulture Engineering & Technology Center, Shenyang Agricultural University, No. 120, Dongling Road, Shenyang 110866, China

## Abstract

Drought induces tomato (*Solanum lycopersicum*) flowers and fruits drop, which causes serious yield and economic losses in agriculture. However, the mechanism of action remains unclear. N6-methyladenosine (m6A) methylation is a prevalent epigenetic change integral to the growth, development, and adaptation of plants to abiotic stress factors. However, whether it participates in drought-induced abscission remains to be further studied. Here, we report that tomato demethylase alpha-ketoglutarate-dependent dioxygenase B (AlkB) homolog 9B (*SlALKBH9B*) exerts a detrimental influence on the regulation of drought-induced flower drop by mediating ethylene production. We found that drought markedly reduced the expression of *SlALKBH9B*, and knockout of *SlALKBH9B* enhanced flower drop, while overexpression of *SlALKBH9B* delayed the flower drop. Under drought conditions, the ethylene production of *Slalkbh9b* exhibited a considerably greater yield than that of the wild type (WT), while *SlALKBH9B* overexpression plants had lower ethylene production. Application of ethylene could abolish the delayed abscission effect of overexpression of *SlALKBH9B*. Further studies showed that drought downregulated *SlALKBH9B* expression, which specifically enhanced the methylation level of the 3′ untranslated region (UTR) of tomato ethylene excess producer 1 (*SlETO1*), leading to a decrease in the stability of SlETO1 mRNA and its protein translation efficiency. The loss of SlETO1 resulted in the accumulation of tomato 1-aminocyclopropane-1-carboxylic acid synthase 3 (SlACS3) and SlACS8 in the abscission zone (AZ) and then boosted ethylene production to accelerate abscission. Our results show that SlALKBH9B is an important inhibitor for drought-induced abscission and reveal a new mechanism through which drought-enhanced ethylene production leads to flower drop.

## Introduction

Abscission is a dynamic physiological process that takes place at specific locations, the abscission zones (AZs) [1, 2]. Drought causes the severe abscission of flowers and fruits, leading to serious yield and economic loss in agriculture [3]. Ethylene plays a dominant role in positively controlling abscission in many plants [4, 5]. Although many studies have reported that drought induces an increase in ethylene production to promote abscission, the related mechanisms still need to be further studied.

In plants, the enzyme 1-aminocyclopropane-1-carboxylic acid (ACC) synthase (ACS) transforms S-adenosyl-L-methionine (SAM) into ACC, which is then oxidized by ACC oxidase (ACO) to produce ethylene. Generally, the ACS-catalyzed reaction is a rate-limiting step [6–8]. In order to limit the synthesis of ethylene, posttranslational modulation of ACS is essential [8]. Kinases, phosphatases, and ubiquitin-proteasome systems all control the stability of ACS proteins, which are key to ethylene production in response to stress and throughout development. Based on their regulatory mechanisms, there are three main categories of ACS proteins: Phosphorylation motifs recognized by both MAPK (Mitogen-activated protein kinase) and CDPK (Calcium-dependent protein kinase) are present in type I proteins, and these regulatory sites also being identified in type I isoforms, CDPK and E3 ligase have target sites in type II, while these regulatory proteins have no target sites in type III [9]. Type I ACS is stably phosphorylated and promotes ethylene production [10–12]. Dephosphorylation, on the other hand, has been demonstrated to accelerate ACS degradation, although this varies depending on the kind of ACS [13, 14]. E3 ubiquitin ligases ethylene overproducer 1 (ETO1) and ETO1-Like (EOL) interact with type II ACSs and result in ubiquitination and degradation of type II ACSs by the 26S proteasome, decreasing ethylene production [15, 16]. In litchi (*Litchi chinensis* Sonn.), exogenous application of brassinosteroids can inhibit the expression of ethylene biosynthesis genes *LcACS1/4* and *LcACO2/3* in the fruit AZ, preventing ethylene-induced fruit drop [17]. However, the role of ACS stability in drought-induced abscission has been less studied.

One of the most prevalent RNA modifications on mRNA is the N6 position methylation of adenosine nucleotides, which is known as N6-methyladenosine (m6A) modification. m6A modification is capable of regulating gene expression across a diverse range of eukaryotes [18]. According to reports, m6A modification exerts a vital function in plants’ growth and development, together with their reactions to biotic and abiotic stressors, as well as the improvement of crop traits [19, 20]. m6A is a reversible and dynamic modification [21], which is installed, removed, and recognized by methyltransferase (writer), demethylase (eraser), and m6A binding protein (reader), respectively [22–25]. A complex regulatory system made up of writers, erasers, and readers directs the creation, removal, and decoding of m6A. Demethylase maintains the dynamic balance of m6A modification levels within cells by demethylating the bases modified by m6A modification [26]. Currently, m6A methylation’s effect on crops’ mRNA metabolism has improved significantly, mainly in two areas: mRNA degradation and translation [27]. The m6A modification located in the stop codon or the 3′ untranslated region (UTR) has been proven to have a negative regulatory effect on the mRNA abundance of tomato fruits [28] and strawberry fruits [29]. Conversely, the m6A enriched in the CDS region in mature strawberry fruits tended to positively regulate mRNA levels [29]. The different effects of these m6A modifications during growth and development on mRNA abundance confirmed that m6A can affect mRNA stability [29, 30]. The deposition of m6A around the stop codon or within the 3′ UTR region can reduce mRNA stability, whereas m6A in the CDS region can promote mRNA stability. Furthermore, the impact of m6A abundance on translation efficiency has been confirmed in multiple crops. For instance, in corn seedlings, m6A deposition in different transcripts exhibits distinct effects on translation efficiency. Specifically, low m6A enrichment near the start codon or within the 3′ UTR tends to enhance mRNA translation, whereas excessive m6A deposition within the 3′ UTR reduces translation efficiency [31]. In strawberry fruits and apple leaves, m6A methylation has also been shown to promote mRNA translation [29, 30].

*ALKBHs*, the homologous demethylase genes of human *FTO,* [32] and *AlkB* [33], are essential in the m6A demethylation reaction under drought stress. The *Arabidopsis* (*Arabidopsis thaliana*) genes *ALKBH8B* and *ALKBH10B* act as opposite regulators of abscisic acid (ABA) signaling when the plant is under salt stress [34, 35]. The m6A modification in the *GhALKBH10B* mutant enhanced the drought resistance of cotton by improving the mRNA stability of genes associated with the signaling pathway, ABA production, and Ca^2+^-related processes [36]. Maize demethylase genes *ALKBH10A* and *ALKBH10B*, as well as *HrALKBH10B*, *HrALKBH10C*, and *HrALKBH10D* in sea-buckthorn (*Hippophae rhamnoides Linn*), were also significantly upregulated by drought stress. This suggests that these two plant species may enhance their adaptation to drought stress by decreasing m6A levels [37, 38]. In *Arabidopsis* and rice (*Oryza sativa*), upregulated *AtALKBH6* and *OsALKBH1*, and downregulated *OsALKBH6*, *OsALKBH8B*, and *OsALKBH10A*, were also found in response to drought stress [39, 40]. Overexpression of the ALKBH homologous protein of demethylase FTO also enhanced rice and potato (*Solanum tuberosum*) resistance to drought [41]. However, it has to be seen whether *ALKBHs* generate some contributions to drought-induced abscission, as well as the underlying regulatory mechanism.

In this study, we investigated how the demethylase SlALKBH9B affects tomato flower drop caused by drought. A gene called SlETO1 that influences ethylene production was eliminated via the application of m6A-seq analysis. The methylation enrichment level in the 3′ UTR of SlETO1 is significantly decreased by SlALKBH9B. Thus, enhancing SlETO1's mRNA level and protein translation efficiency. In the final stages, it induced SlETO1 to degrade the type II SlACS3 and SlACS8 proteins, which decreased flower pedicel AZ's ethylene production and inhibited flower dropping. Our findings provide insight into how demethylases prevent drought-induced flower loss by affecting the release of ethylene.

## Results

### *SlALKBH9B* negatively regulates abscission in response to drought stress

**Figure 1:**
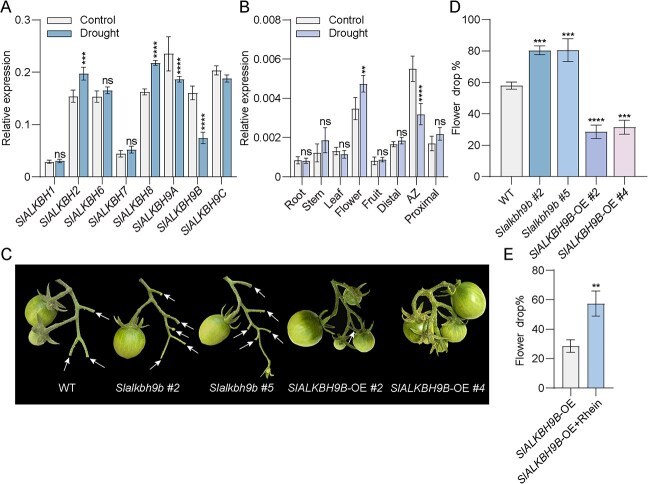
Demethylase *SlALKBH9B* negatively regulates tomato flower drop under drought conditions. (**A**) The relative expression of eight *SlALKBH* genes in tomato AZ of WT plants was analyzed using RT-qPCR under both normal and drought conditions. The data is the mean of three replicates ± standard deviation (SD). Significant differences were identified through a two-way ANOVA employing Šídák’s test: ns, no significant difference; ^***^P < 0.001; ^****^P < 0.0001. Among these genes, those presented in red font signify genes exhibiting significant differential expression. (**B**) The expression levels of *SlALKBH9B* in various parts of tomato plants under normal and drought conditions. Values are means ± SD from 3 biological replicates. Different * numbers indicate significant differences, as assessed by two-way ANOVA with Šídák’s test, ns, no significant difference; ^****^P < 0.0001. (**C**) The representative inflorescence fruit phenotypes of WT, *Slalkbh9b #2*, *5*, and *SlALKBH9B-*OE *#2*, *4* under drought conditions. Arrows indicate flowers that have fallen off. Scale bar: 1 cm. (**D**) Flower drop percentages of WT, *Slalkbh9b*, and *SlALKBH9B*-OE lines during drought conditions. The values represent means ± SD derived from three biological replicates. Significant differences were identified using one-way ANOVA with Šídák’s test: ns, no significant difference; ^***^P < 0.001; ^****^P < 0.0001. (**E**) Proportion flowers that dropped off *SlALKBH9B*-OE plants after Rhein treatment under drought conditions. Data are the mean ± SD of three biologically independent trials. The determination of significant differences was done using the *t*-test (two-tailed): ^**^P < 0.01.

The tomato genome contains eight *ALKBH* genes (Supplementary Fig. S1). To investigate the potential function of m6A demethylase ALKBHs in drought-induced flower drop, we initially analyzed their expression profiles under drought stress. During drought conditions, *SlALKBH2* and *SlALKBH8* expression levels were upregulated, but *SlALKBH9A* and *SlALKBH9B* expression levels were downregulated (Fig. 1A). Additionally, we found that *SlALKBH9B* was significantly downregulated only in the AZ of the flower pedicel under drought conditions (Fig. 1B). The expression of *SlALKBH9B* under drought was downregulated by more than 2-fold difference compared with the control, which was of biological significance. We then used virus-induced gene silencing (VIGS) to suppress *SlALKBH9B* expression (Supplementary Fig. S2A and E). Under drought conditions, 73.5%, 75.93%, and 74.92% of the flowers in pTRV2-*SlALKBH9B #1*, pTRV2-*SlALKBH9B #5*, and pTRV2-*SlALKBH9B #8* plants had dropped, respectively, compared to 59.73% of the flowers of pTRV2-empty vector plants. In addition, we also utilized the VIGS assay to conduct functional verification on the remaining *SlALKBH* genes whose expression levels changed significantly due to drought. The other three genes silenced plants had no significant differences in flower drop rate (Supplementary Fig. S2B–D and F–H). We used genome editing mediated by clustered regularly interspaced short palindromic repeats (CRISPR)/CRISPR-associated nuclease 9 (Cas9) to create knockout *SlALKBH9B* mutants to gain a clearer insight into the role of *SlALKBH9B* (Supplementary Fig. S3A). In drought conditions, 57.96% of wild-type (WT) flowers dropped before fruit set, while *Slalkbh9b #2* plants had 80.37% and *Slalkbh9b #5* had 80.56% (Fig. 1C and D). The overexpression Sl*ALKBH9B* lines were generated through *Agrobacterium tumefaciens*-mediated transformation. The two lines of *SlALKBH9B* (*SlALKBH9B-*OE *#2* and *SlALKBH9B-*OE *#4*) were selected for additional research because their expression levels were 6.1- and 5.7-fold higher, respectively, than those in the WT plants (Supplementary Fig. S3B). Under drought, the flower drop rates of *SlALKBH9B*-OE *#2* and *SlALKBH9B-*OE *#4* were 28.54% and 31.38%, respectively (Fig. 1C and D). Meanwhile, we detected the flower drop rates of *SLALKBH9B*-OE and *Slalkbh9b* under well-watered conditions. The results showed that there was no significant difference in the flower drop rates of these two lines under well-watered conditions compared with WT (Supplementary Table S1). In addition, we observed that *SlALKBH9B* was highly expressed in flowers under drought conditions (Fig. 1B). Therefore, we observed the phenotype of senescence of tomato flower by referring to the article published by LOP-Tous [42]. The results suggested *Slalkbh9b* significantly accelerated flower senescence compared with the WT (Supplementary Table S2). The Rhein was selected for test since it inhibits alkb m6A demethylase activity [43]. Rhein treatment was found to significantly enhance the flower drop in *SlALKBH9B*-OE tomato plants compared to plants without Rhein treatment (Fig. 1E). Those results indicated that *SlALKBH9B-*dependent N^6^-methyladenosine played a negative role in flower drop triggered by drought.

### *SlALKBH9B* inhibits ethylene production in tomato flower pedicel

**Figure 2:**
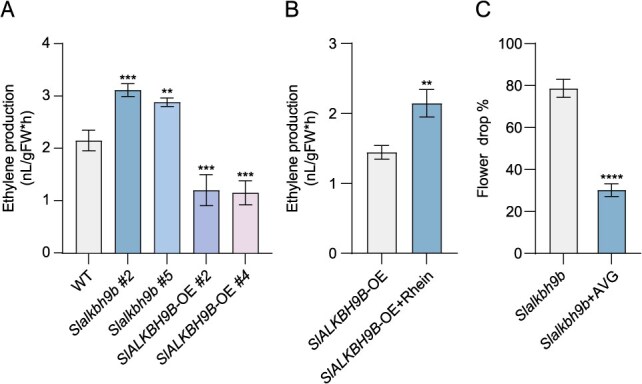
*SlALKBH9B* inhibits ethylene production in tomato flower pedicels. (**A**) AZ from WT, *Slalkbh9b*, and *SlALKBH9B-*OE plants, where ethylene production was determined after drought. Data are the average ± SD from three separate experiments in each row (Significant differences were evaluated through one-way ANOVA with the application of Šídák’s test: ns, no significant difference; ^**^P < 0.01; ^***^P < 0.001). (**B**) The ethylene production was determined after being treated with Rhein under drought conditions. The data represent the mean ± SD from three separate experiments for each line. ^**^P < 0.01 (two-tailed *t*-test). (**C**) Under drought conditions, *Slalkbh9b* was treated with AVG to assay flower drop. Data represent the mean ± SD from three separate experiments for each line (*t*-test): ^****^P < 0.0001).

Drought can boost ethylene production to modulate plants’ response to drought [44–46]. Ethylene is a master regulator for various organ abscission [4]. We explored whether *SlALKBH9B* regulates abscission via ethylene, and assessed ethylene production from the pedicels AZ of WT, *Slalkbh9b*, and *SlALKBH9B-*OE plants under drought conditions (Fig. 2A). Compared to WT, *SlALKBH9B-*OE shows significantly lower ethylene production, whereas *Slalkbh9b* shows higher ethylene production. Moreover, we measured ethylene production from the *SlALKBH9B-*OE pedicel AZ with or without Rhein treatment under drought conditions. The findings demonstrated that the *SlALKBH9B-*OE plants treated with Rhein had noticeably more ethylene production than the control plants (Fig. 2B). Aminoethoxyvinylglycine (AVG) functions as an inhibitor of the enzyme ACC synthase, which is vital for the biosynthesis of ethylene [47]. Then we treated *Slalkbh9b* plants with 10 mM AVG under drought conditions and measured the flower drop rate until fruit set. It was found that there was a significant decrease in flower drop in *Slalkbh9b* after being treated with AVG (Fig. 2C). These results indicated that *SlALKBH9B* delayed drought-induced abscission mainly by negatively regulating the biosynthesis of ethylene.

### Variations in methylome between *Slalkbh9b* and WT

**Figure 3:**
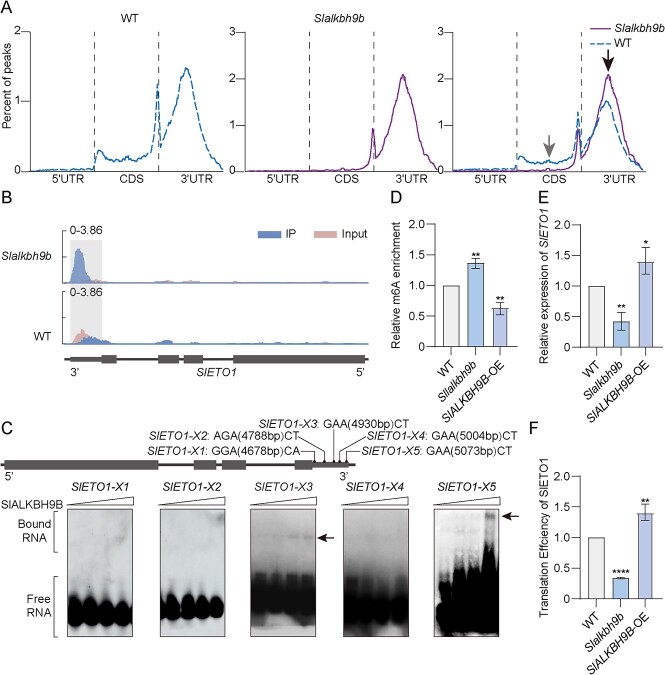
SlALKBH9B demethylates m6A in the mRNAs of negative regulators of ETH production. (**A**) Distribution of m6A peaks in the 5′ UTR, CDS, and 3′ UTR of the transcript fragments. The gray arrow indicates reduced peak enrichment of *Slalkbh9b* in the CDS region compared to WT, while the black arrow indicates increased peak enrichment in the 3′ UTR region. (**B**) Levels of m6A modification of *SlETO1* in AZ of *Slalkbh9b* and WT plants. (**C**) Electrophoretic mobility changes were observed of purified recombinant SlALKBH9B protein (0, 4, 8, and 12 μM) and with increasing concentrations of RNA substrate (10 pmol). The bound probes are indicated by black arrows. (**D**, **E**) Results of m6A-IP-qPCR show relative m6A levels (**D**), and RT-qPCR (**E**) displays the comparative expression levels of *SlETO1* in WT, *Slalkbh9b*, and *SlALKBH9B*-OE pedicel AZ. Results are expressed as the mean ± SD of three biological replicates (One-way ANOVA with Šídák’s test: ns, no significant difference; ^*^P < 0.05; ^**^P < 0.01). (**F**) Translational efficiency of SlETO1 in *Slalkbh9b*, *SlALKBH9B*-OE, and WT plants under drought conditions. (One-way ANOVA with Šídák’s test was used to identify significant differences: ^**^P < 0.01; ^****^P < 0.0001).

To explore whether the m6A methylation pattern in *Slalkbh9b* mutants might be different from that in WT under drought conditions, we performed m6A-seq (deep transcriptome sequencing of m6A-modified RNA) on *Slalkbh9b* and WT AZ under drought treatment. We identified 13 966 and 10 681 confidence m6A peaks in WT and *Slalkbh9b*, respectively. There were 7563 different peaks between WT and *Slalkbh9b*. In comparison with the WT plants, as many as 6083 transcripts in *Slalkbh9b* showed elevated m6A levels, while only 1480 transcripts displayed reduced m6A enrichment (Supplementary Fig. S4A). According to the reference annotation, each transcript was separated into five non-overlapping segments: the 3′ untranslated region (UTR), the coding sequence (CDS), the transcription termination site (TTS), the stop codon, and the 5′ UTR. The distributions of m6A peaks in the WT and *Slalkbh9b* plants were analyzed. Across all samples, m6A peaks were primarily found around the 3′ UTR and the CDS (Supplementary Fig. S4B). Furthermore, analysis was conducted on the m6A peak distributions in both WT and *Slalkbh9b* plants. Compared to the WT plants, which had 34.81%, the *Slalkbh9b* plants had a significantly lower m6A peak proportion of 2.07% in the CDS region. Similarly, the percentages in the TTS region (0.42% vs 2.03%) and the 5′ UTR (0.33% vs 1.97%) were also lower than those in the WT plants. In contrast, the percentages in the stop-codon region (34.77% vs. 16.97%) and the 3′ UTRs (62.41% vs. 44.22%) were higher. A majority of gene transcripts with m6A modification had a single m6A peak (86.87%), while some had two peaks (11.51%), and only a small number had three or more peaks (1.62%) (Supplementary Fig. S4C), similar to levels found in *Arabidopsis* and tomato [28, 48, 49]. Furthermore, hypergeometric optimization of motif enrichment (HOMER; http://homer.ucsd.edu/homer/) was utilized to discover the sequence motifs enriched within the m6A peaks in tomato [50]. The clustering of m6A peaks by HOMER revealed the ‘UGUAUA’ sequence motif in tomato (Supplementary Fig. S44D), similar to that in *Arabidopsis* [51] and maize (*Zea mays L*.) [37]. As shown in Fig. 3A, there was a significant enrichment of m6A modifications around the CDS and within the 3′ UTR. The distribution of *Slalkbh9b* in the CDS region is significantly decreased (shown by gray arrow) compared to WT, and the distribution of *Slalkbh9b* in the 3′ UTR region is significantly increased (shown by black arrow). Considering *SlALKBH9B* has demethylase activity, we focused on analyzing the genes that were significantly upregulated in the 3′ UTR region. The KEGG analysis indicated these genes were mainly classified in the mRNA surveillance pathway, endocytosis, phagosome, and the N-Glycan biosynthesis (Supplementary Fig. S5).

### *SlALKBH9B* demethylates m6A in the mRNAs of negative regulators of ethylene production

Among those m6A hypermethylated transcripts, a negative regulator of ethylene production, ETHYLENE OVERPRODUCER1 (ETO1), a homologous gene to *AtETO1*, attracted our attention. Lacking ETO1 displays an ethylene overproduction phenotype [52]. *Arabidopsis* ETO1 acts as an inhibitor of ethylene synthesis by facilitating the proteasomal degradation of ACS (ACS4/5/9), hence reducing ethylene production [15]. ETO1 holds a significant position in drought [53], salt [54], and heavy metal stress [53]. We detected elevated m6A peaks in the 3′ UTRs of *SlETO1* in the *Slalkbh9b* as opposed to the WT under drought conditions (Fig. 3B), implying that *SlETO1* is a potential target of SlALKBH9B. The presence of m6A modification at the stop codon or 3′ UTR regions was found to decrease mRNA abundance in tomato fruit [55], maize seedlings [56], and strawberry (*Fragaria vesca*) fruit [29]. We then constructed a *SlALKBH9B*-His vector and purified it from *Escherichia coli* BL21. To do in vitro RNA electrophoretic mobility change analysis (EMSA) with 42-mer RNA synthesized in vitro with m6A. The EMSA findings indicated that SlALKBH9B directly binds to the m6A-modified *SlETO1* 3′ UTR m6A sites at *X3* and *X5* (Fig. 3C). Using m6A-IP followed by quantitative PCR (m6A-IP-qPCR), we verified that *SlETO1* mRNA is highly m6A-methylated in *Slalkbh9b* and has reduced m6A methylation in *SlALKBH9B*-OE lines under drought conditions compared to the WT (Fig. 3D). Furthermore, m6A modification occurring in proximity to the stop codon or within the 3′ UTR segment has been shown to decrease transcript stability [29, 56]. Consistent with the expected results, qRT-PCR assay demonstrated that *SlETO1* transcription levels in the *Slalkbh9b* and *SlALKBH9B*-OE pedicel AZ were significantly down- and up-regulated, respectively, in comparison to those of WT (Fig. 3E), implying that m6A deposition in the 3′ UTR region was inversely linked with mRNA abundance. In addition, we examined the protein translation efficiency of SlETO1 in WT, *Slalkbh9b*, and *SlALKBH9B-*OE plants. The experimental results demonstrated substantial alterations in SlETO1 protein synthesis rates, showing a marked reduction in *Slalkbh9b* while exhibiting a notable elevation in *SlALKBH9B*-OE transgenic plants relative to WT (Fig. 3F).

### SlETO1 works downstream of SlALKBH9B and negatively regulates flower drop in reaction to drought stress

**Figure 4:**
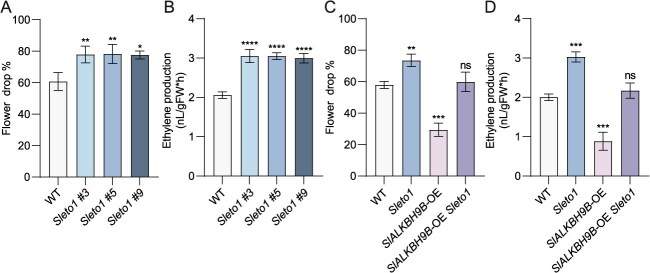
*SlETO1* suppresses abscission under drought conditions and acts genetically downstream of SlALKBH9B. (**A**) and (**B**) WT and *Sleto1* lines’ percentage of flower drop and ethylene production under drought conditions. The value is the average of the results of three times (One-way ANOVA with Šídák’s test: ns, no significant difference; ^*^P < 0.05; ^**^P < 0.01; ^****^P < 0.0001). (**C**) and (**D**) Ethylene production of the WT, *SlALKBH9B*-OE, *Sleto1*, and *SlALKBH9B*-OE *Sleto1* lines as a percentage of flower drop during drought. Three biological replicates’ means ± SD make up the values. Using Šídák’s test, one-way ANOVA: ns, no significant difference; ^**^P < 0.01; ^***^P < 0.001).

To investigate the biological role of SlETO1 in flower drop triggered by drought conditions, we first detected the expression of *SlETO1* in various parts of tomato plants under drought and well-watered conditions. The results showed that SlETO1 is significantly downregulated only in AZ under drought conditions (Supplementary Fig. S6). Then, we constructed the CRISPR/Cas9 plant of *SlETO1* (Supplementary Fig. S7) and tested the flower drop and ethylene production under drought conditions. The flower drop rate of the *Sleto1* cultivars was considerably higher than that of the WT (Fig. 4A). Likewise, ethylene production in the pedicel AZ of *Sleto1* was notably greater than in WT (Fig. 4B). Later, we crossbred *SlALKBH9B-*OE with *Sleto1* to obtain *SlALKBH9B-*OE *Sleto1* F2 generation and assayed ethylene production and flower drop rate under drought conditions. The figure shows that the flower drop rate and ethylene production of *SlALKBH9B-*OE *Sleto1* were similar to WT (Fig. 4C and D). The above genetic data indicated that SlETO1 acts downstream of SlALKBH9B to regulate ethylene production during drought-induced flower drop. Meanwhile, we found that *SlETO1* is expressed in flowers besides AZ (Supplementary Fig. S6). We also observed flower senescence in the WT and *Sleto1*. The results showed that, similar to *SlALKBH9B*, *Sleto1* significantly accelerated flower senescence (Supplementary Table S3).

### SlACS3 and SlACS8 protein accumulation are affected by SlETO1 under drought

**Figure 5:**
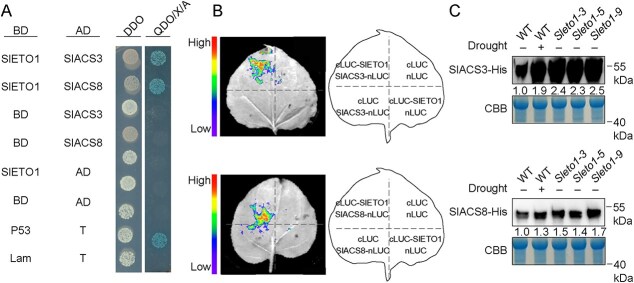
Protein stability of SlACS3 and SlACS8 is regulated by SlETO1. (**A**) Through the yeast two-hybrid experiment proved SlETO1 interacts with SlACS3 and SlACS8. (DDO indicates a medium with a double deficiency of tryptophan and leucine (SD/−Trp/−Leu), and QDO refers to a medium with a quadruple deletion of tryptophan, leucine, histidine, and adenine (SD/−Trp/−Leu/−His/−Ade)). (**B**) The interaction between SlETO1 and SlACS3 or SlACS8 in tobacco leaves is shown through LUC assays. (**C**) Recombinant SlACS3-His and SlACS8-His underwent cell-free degradation assays. These purified proteins were mixed with total protein extracts from the AZ of WT and Sleto1, with or without exposure to drought. On the gel with Coomassie brilliant blue (CBB), staining showed the same sample amount.

ETO1/EOLs are part of a CULLIN3 E3 ligase that targets type II ACS isoforms for fast destruction by the 26S proteasome [9]. Previous tests confirm that EOL1 interacts with Arabidopsis types II: ACS2, ACS4, ACS5, and ACS9, but not with other clades, similar to ETO1 [57]. In tomato, SlACS3, SlACS7, and SlACS8 belong to type II ACS, and it was found that only *SlACS3* and *SlACS8* were expressed in the flower pedicel AZ through qRT-PCR, *SlACS3* and *SlACS8* were significantly upregulated by drought, the subsequent experiments were conducted around *SlACS3* and *SlACS8* (Supplementary Fig. S8). To ascertain whether ETO1 in tomato interacts with ACSs, we initially conducted yeast two-hybrid tests to verify that SlETO1 can indeed interact with SlACS3 and SlACS8 (Fig. 5A). Interactions between SlETO1 with SlACS3 and SlACS8 were also confirmed by luciferase (LUC) complementation imaging (LCI) assays (Fig. 5B). We then examined the regulatory mechanism of SlETO1 on type II ACS. We constructed the vectors of SlACS3-His and SlACS8-His, respectively, and induced purification from *E. coli* BL21, and tested their protein stability in WT and *Sleto1* plants with or without drought. The results indicated that the protein stabilities of SlACS3 and SlACS8 are enhanced by drought, and it also increased significantly after *SlETO1* knockout in normal conditions (Fig. 5C).

### *SlACS3* and *SlACS8* are required for drought-induced flower drop

**Figure 6:**
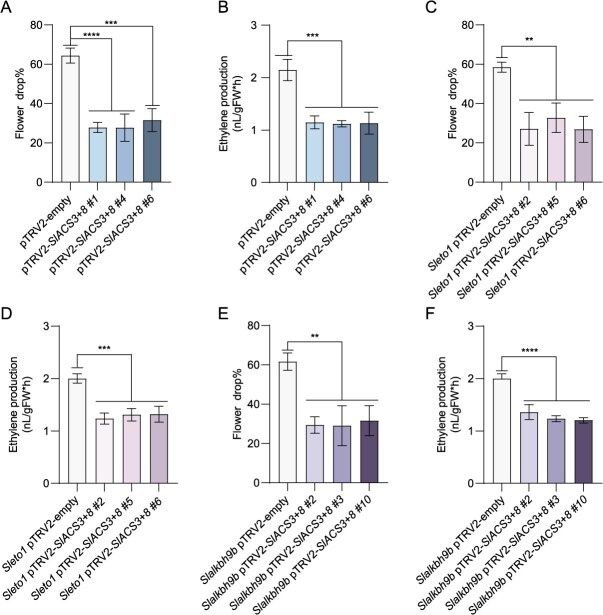
SlACS3 and SlACS8 are located downstream of SlALKBH9B-SlETO1 module during drought-induced tomato flower drop. (**A**) and (**B**) Flower drop and ethylene production of *SlACS3* and *SlACS8* simultaneously silenced lines under drought conditions. (One-way ANOVA with Šídák’s test: ^***^P < 0.001; ^****^P < 0.0001). (**C**) and (**D**) Flower drop percentage and ethylene production in *Sleto1* pTRV2-empty, *Sleto1* pTRV2-*SlACS3 + 8 #1*, *Sleto1* pTRV2-*SlACS3 + 8 #4*, and *Sleto1* pTRV2-*SlACS3 + 8 #6* plants under drought. Three separate biological treatments were used on individual plant lines. Statistical variations are indicated by distinct (*) symbols, analyzed through one-way ANOVA employing Šídák’s correction: ^**^P < 0.01; ^***^P < 0.001. (**E**) and (**F**) Percentage of flower drop and ethylene production in *Slalkbh9b* pTRV2-empty, *Slalkbh9b* pTRV2-*SlACS3 + 8 #1, Slalkbh9b* pTRV2-*SlACS3 + 8 #4*, and *Slalkbh9b* pTRV2-*SlACS3 + 8 #6* plants under drought. Each plant line underwent three distinct biological treatments. Significant differences are marked by different (*) symbols, as identified using one-way ANOVA with Šídák’s test: ^**^P < 0.01; ^****^P < 0.0001.

To further explore *SlACS3* and *SlACS8* roles in drought-induced ethylene production and flower drop. We assessed the flower drop rate and ethylene production after silenced expression of *SlACS3* and *SlACS8* by VIGS. It was discovered that down-regulating *SlACS3* or *SlACS8* decreased ethylene production and flower drop rate (Supplementary Fig. S9A–F). While *SlACS3* and *SlACS8* simultaneously silenced plants further decreased ethylene production and flower drop rate (Supplementary Fig. S9G and Fig. 6A and B). To test the genetic relationship between *SlALKBH9B*, *SlETO1*, *SlACS3*, and *8*, we silenced *SlACS3* and *SlACS8* in *Slalkbh9b* and *Sleto1* plants, respectively (Supplementary Fig. S9H and I). These results confirm that SlALKBH9B -SlETO1 modular is located upstream of *SlACS3* and *SlACS8* during drought-induced flower drop (Fig. 6C–F).

## Discussion

### *SlACS3* and *SlACS8* participate in tomato flower drop under drought stress and are key regulators of ethylene production

The phytohormone ethylene plays a crucial regulatory role in the abscission processes of foliar organs, floral structures, and reproductive tissues in vegetation [58–60], and also plays an important role in the regulation of drought stress responses [61]. Different *ACS* genes in plants can be specifically induced when the plants are subjected to different abiotic stressors. In trifoliate orange (*Poncirus trifoliata*), ethylene can enhance drought tolerance by promoting root hair formation and increasing photosynthetic efficiency [62]. OsERF109 hinders ethylene production by negatively impacting the expression of genes *OsACS6* and *OsACO2* associated with ethylene synthesis, and it also diminishes drought resilience in rice [63]. Under cadmium (Cd) stress, the transcription of *ACS2* and *ACS6* increases, promoting ethylene production and enhancing the stress resistance of *Arabidopsis* plants [64]. Drought-induced upregulation of *RhACS1* and *RhACS2* in rose (*Rosa hybrida*) further leads to increased ethylene synthesis, and promoters of these genes specifically respond to abiotic stress [65]. As mentioned above, numerous studies have demonstrated that ethylene plays an essential role under drought conditions. However, its mechanism in drought-induced tomato flower drop still requires further exploration. This study revealed that the expressions of *SlACS3* and *SlACS8* were upregulated under drought stress (Supplementary Fig. S8). Silencing both *SlACS3* and *SlACS8* further decreased ethylene production and the flower drop rate (Fig. 6A and B). In conclusion, *SlACS3* and *SlACS8* are key factors contributing to drought-induced tomato flower drop.

### The stability of SlACS3 and SlACS8 proteins is regulated by SlETO1.

ACS type II can be degraded through ubiquitination mediated by ETO1, EOL1, and EOL2 [66]. Type II ACS5 in *Arabidopsis* can form an interaction with ETO1, which is responsible for encoding an E3 ligase component that includes a BTB domain. This association allows ETO1 to selectively target ACS5 for degradation [15]. The *Ateto1* caused ethylene production to exceed that of the WT by nearly 10-fold [67]. ETO1 and EOL1 have been found to enhance drought resistance in rice and *Arabidopsis* [53]. This study found that SlETO1 can engage with type II SlACS3 and SlACS8 to trigger their breakdown, aligning with findings in *Arabidopsis* (Fig. 5A–C). Furthermore, we found that *SlETO1* expression was significantly decreased under drought (Supplementary Fig. S6). When *SlETO1* was knocked out, the rate of tomato flower drop and the ethylene production of the flower pedicel AZ were substantially elevated in contrast to the WT under drought conditions (Fig. 4A and B). This shows that losing SlETO1 function is vital for abscission when drought occurs. Silencing *SlACS3* and *SlACS8* in *Sleto1* significantly decreased flower drop and flower pedicel AZ ethylene production compared with *Sleto1* pTRV2-empty under drought conditions (Fig. 6C and D), confirming that SlETO1 acts upstream of SlACS3 and SlACS8 during drought-induced tomato flower drop.

### SlETO1 negatively regulated flower drop under drought and was regulated by *SlALKBH9B*

As the predominant internal RNA alteration in eukaryotic transcripts, m6A methylation participates in diverse physiological processes, including vegetative growth, developmental regulation, and environmental adaptation mechanisms in plants [68]. Eight *SlALKBH* genes have been discovered in tomato plants. Among them, *SlALKBH9B* show the largest decreased in expression upon drought treatments (Fig. 1A). Furthermore, *SlALKBH9B*-OE and *Slalkbh9b* plants showed significantly lower and higher ethylene production in flower pedicel AZ than the WT, respectively (Fig. 2A). Accordingly, *SlALKBH9B*-OE and *Slalkbh9b* show delayed and accelerated flower drop, respectively, indicating that *SlALKBH9B*-dependent m6A methylation negatively influences drought-induced flower abscission by modulating ethylene production.

Research into m6A methylation has revealed its significant roles in the regulation of hormone responses. m6A methylation can regulate the ripening of strawberry fruits through the ABA pathway [55]. *ALKBHs* have been reported in *Arabidopsis* as demethylases that remove m6A modifications [25]. m6A-seq assays demonstrated that the degree of methylation of the 3′ UTR region in *Slalkbh9b* was elevated compared to the WT under drought conditions (Fig. 3A). After screening, a potential key ethylene production gene, *SlETO1*, was selected. The results also confirmed that the methylation level of *SlETO1* in the 3′ UTR region of *Slalkbh9b* was significantly higher than that in the WT (Fig. 3D). The EMSA confirmed that SlALKBH9B could bind to the *SlETO1-X3* and *SlETO1-X5* sites in the 3′ UTR region of SlETO1 (Fig. 3C). These results indicated that SlALKBH9B regulated the methylation of the 3′ UTR of SlETO1 by directly binding it. Previous studies have reported that methylation enrichment in the 3′ UTR region decreases mRNA abundance and protein translation efficiency [69]. In this investigation, *Slalkbh9b* exhibited a higher m6A modification at the 3′ UTR of *SlETO1* than WT plants, but the transcription abundance and translation efficiency of SlETO1 were notably reduced in *Slalkbh9b* than in the WT plants (Fig. 3D–F), suggesting that SlALKBH9B-dependent post-transcriptional regulation of SlETO1 is essential for its function.

We crossed *Sleto1* and *SlALKBH9B*-OE to obtain *Sleto1 SlALKBH9B-*OE lines. The flower drop and ethylene production of the flower pedicel AZ measured under drought conditions were similar to those of WT, but significantly lower than that in *Sleto1* (Fig. 4C and D). These results indicated that SlETO1 is not the only downstream factor of SlALKBH9B. Then, when we silenced *SlACS3* and *SlACS8* in the *Slalkbh9b*, there was a significant decrease in flower drop and pedicel AZ ethylene production compared with the *Slalkbh9b* under drought conditions (Fig. 6E and F). In conclusion, SlALKBH9B regulates tomato flower drop through the SlETO1-SlACS3 and SlACS8 modules under drought conditions. We examined *SlALKBH9B* and *SlETO1* expression in other areas, it was found that *SlALKBH9B* was highly expressed in flowers and AZ (Fig. 1B), and *SlETO1* was expressed in the flowers and AZ (Supplementary Fig. S6). Then, we investigated whether they had effects on the flower senescence. The results demonstrated that the flower senescence was significantly different between both knockout and overexpression lines when compared to WT (Supplementary Table S2 and S3). Thus, we propose that SlETO1 may be the cause of the effect of SlALKBH9B on flower senescence.

### Conclusion

**Figure 7:**
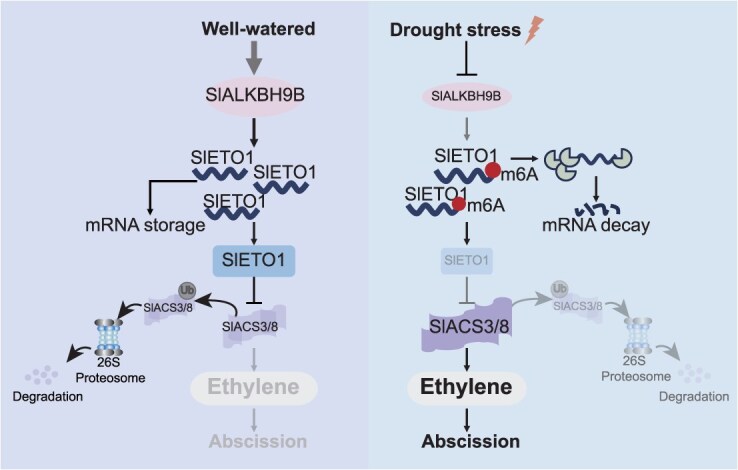
Under drought conditions, the demethylase SlALKBH9B inhibits tomato flower drop by reducing ethylene production. Drought downregulates the expression of *SlALKBH9B*, consequently reducing the demethylation of the downstream region of SlETO1. The enhanced methylation of SlETO1 at the 3′ UTR terminal downregulates its RNA stability and protein translation efficiency. The ubiquitin-mediated degradation of SlACS3 and SlACS8 proteins is reduced when SlETO1 is downregulated. This decrease in degradation results in an increased ethylene production from the tomato pedicel AZ, ultimately causing flower drop.

Here, we analyze a mechanism by which m6A methylation influences tomato flower drop by regulating ethylene production under drought conditions. Under normal conditions, SlALKBH9B in tomato AZ can reduce the methylation level of the 3′ UTR region of SlETO1 through demethylation, and improve the RNA stability and translation efficiency of SlETO1. SlETO1 causes the degradation of SlACS3 and SlACS8 proteins, thus maintaining a minimal concentration of ethylene production in the flower pedicel AZ. The *SlALKBH9B* expression is reduced under drought conditions, causing an increase in methylation of the 3′ UTR of SlETO1 and hence impairing its function. Subsequently, SlACS3 and SlACS8 proteins accumulated to boost ethylene production, which promotes tomato flower drop (Fig. 7).

## Materials and methods

### Cultivation parameters and experimental treatments for plants

Tomatoes of the ‘Ailsa Craig (AC)’ cultivar (*Solanum lycopersicum*) were utilized as the WT and as the genetic foundation for producing transgenic plants. The *SlALKBH9B*-OE, *Slalkbh9b*, and *Sleto1* lines were generated by Biogle Biotechnology (Hangzhou, China). Plants were raised in a greenhouse environment with 16 hours of light at 25°C and 8 hours of dark at 15°C. For the drought treatment, either WT or mutants were planted in well-watered soil, waited around eight weeks to grow, and then planted and treated with drought. The drought treatment is based on earlier findings and has been slightly altered [70, 71]. To put it briefly, control plants received daily morning watering until their soil water content (SWC) reached 70%. SWC was kept at 30% for drought treatment, and it was readjusted each morning. In order to ensure that the transgenic and WT plants remained at the same degree of drought, the relative water content (RWC) in their leaves was calculated. The method for calculating RWC is (fresh weight-dry weight) × 100/(turgid weight-dry weight) [72]. For Rhein treatment, Rhein solution (20 μg/mL) was sprayed onto the AZ of drought-treated plants. Similarly, the AVG treatment (10 mmol) was directly applied to the AZ of drought-treated plants.

The growth chamber (model LPH-350S, NK system, Japanese origin) was used to grow *Nicotiana benthamiana* plants. The environmental conditions were stictly controlled as follows: a diurnal temperature cycle of 25°C during the day and 18°C at night, a photoperiod characterized by 16 hours of light followed by 8 hours of darkness, a fixed relative humidity of 70% along with a photosynthetic photon flux density (PPFD) of 300 μmol∙m^−2^∙s^−1^.

Liquid nitrogen was employed to quickly freeze the plant samples. Immediately after freezing, the samples were then stored at −80°C until they were ready for examination.

### Survey of abscission phenotypes

From the inflorescence stage, we maintained drought conditions until fruit set, calculated the drop rates of WT and mutant plants under drought conditions, and ensured that each cluster contained at least five inflorescences per cluster. The drought treatment was carried out as described above.

### Ethyene production measurements

Fifteen flower pedicels were collected from each experiment, and a minimum of three biological replicates were examined. After drought treatment, the flower pedicel AZ samples of the treatment group, the untreated group, and each mutant line were taken out and treated in 50 mM phosphate buffer solution with pH 7.4 for 10 minutes to eliminate the effect of trauma on ethylene production [73]. Then the quality was detected and subsequently transferred to a 10 mL sealed glass container, which was maintained at room temperature for 60 minutes [73–75]. Then, the gas was drawn from the sealed jar using a 1 mL syringe. The ethylene production test has been quantified as previously mentioned, the gas was analyzed by gas chromatography using a Varian GC-3800 equipped with a GDX-102 column (Dalian Institute of Chemical Physics, China), with nitrogen (20 mL∙min^−1^) as the carrier gas [74, 75].

### RNA extraction and RT-qPCR analysis

An RNA extraction kit (AG, Hu Nan, China) was employed to extract RNA from the pedicel AZ of each line. Subsequently, 5 × Evo M-MLV RT Master Mix* was used to reverse-transcribe 1 μg of RNA into the first-strand cDNA (AG, Hunan, China). Gene-specific primers and a 2 × SYBR Green Pro Taq HS Premix (AG, Hunan, China) reaction system were used to perform quantitative real-time polymerase chain reaction (RT-qPCR). The *ACTIN* gene (NCBI: NM_00133011.1) from tomato served as an internal control for quantitative normalization.

### VIGS

The Sol Genomics Network (https://vigs.solgenomics.net/) was utilized in the VIGS tools to create fragments of *SlALKBH2*, *SlALKBH8*, *SlALKBH9A*, *SlALKBH9B*, *SlACS3*, *SlACS8*, and *SlACS3 + 8* that are specific to coding sequences. And these fragments were connected to the pTRV2 vector through PCR amplification. *Agrobacterium tumefaciens* GV3101 (Sangon Biotech (Shanghai) Co., Ltd., Shanghai, China) received transformations of pTRV2 constructs with the gene fragments and pTRV1. VIGS experiments were performed on tomato stems following a previously described silencing method [76]. Fifteen days after infection, the silencing efficiency was detected by RT-qPCR. The primers used in this study are listed in Supplementary Table S5.

### Yeast two-hybrid (Y2H) experiment

*SlETO1*’s whole coding sequence was linked to pGBKT7 after being amplified by PCR. (Clontech, Beijing, China). Following PCR amplification, the complete coding sequences of S*lACS3* and *SlACS8* were subcloned into pGADT7 (Clontech, Beijing, China) via restriction enzyme digestion and ligation, respectively. The above coding sequences were amplified using the cDNA template derived from fruit 10 days after color breaking. Using the Matchmaker Golden Yeast two-hybrid (Y2H) system, the Y2H experiment was conducted (Clontech, Beijing, China). The primers utilized in these amplifications are included in Supplementary Table S5. To detect potential protein–protein interactions, the His Ade reporter genes were employed in the presence of 5 mM Aureobasidin A (Aba) and 100 μg/mL 5-Bromo-4-chloro-3-indolyl β-d-galactopyranoside (X-gal) (Coolaber, Beijing, China). This setup allowed for the identification of positive interactions based on the growth and color changes of the yeast colonies.

### LUC assay

For the interaction between SlETO1 and SlACS3, as well as SlETO1 and SlACS8, the *SlETO1* fragments were inserted into pCAMBIA1300-cLUC. Separately, the *SlACS3* and *SlACS8* coding sequences were included in pCAMBIA1300-nLUC. The primers for these insertions can be found in Supplementary Table S5. Each GV3101 strain (Sangon Biotech (Shanghai) Co., Ltd., Shanghai, China) containing the designated constructs was cultured separately until reaching an OD_600_ of 0.8. Then resuspended in an infiltration buffer containing specific components to OD_600_ = 1.0 [71]. Equal volumes of the adjusted cultures of different strains were then mixed. Before penetrating the tobacco leaves, an appropriate Agrobacterium cell solution was combined at a volume ratio of 1:1, as was previously mentioned [77]. The activities of firefly luciferase were measured using the NightSHADE LB 985 In Vivo Imaging System (Berthold Technologies, https://www.berthold.com/en/).

### Protein stability analysis

The pET30a vector was applied to individually clone the complete CDS of *SlACS3* and *SlACS8* (Supplementary Table S5). The recombinant vectors generated through molecular cloning were then transformed into *E. coli* Rosetta cells (Sangon Biotech (Shanghai) Co., Ltd., Shanghai, China). The inducible protein expression was triggered by adding 1 mmol/L IPTG and incubating at 16°C for 16 hours [71]. The recombinant protein was purified using a His-Tag Protein Purification Kit (Beyotime Biotechnology, Jiangsu, China). Subsequently, the protein was identified via immunoblotting with an anti-His antibody (Solarbio, China). Total proteins were extracted from drought-treated WT plants and the AZ of *Sleto1*. The tissue lysis buffer consists of the following components, as mentioned earlier [71]. Each reaction mixture contained 500 μg total protein and 100 ng of the purified proteins SlACS3-His and SlACS8-His. After incubation at 25°C for 30 min, the reaction products were collected, integrated with 4 × protein loading buffer (Coolaber, Beijing, China), and boiled. The protein levels of SlACS3-His and SlACS8-His were determined by immunoblotting.

### Phylogenetic analysis

The sequences of amino acids in ALKBHs from *Arabidopsis* and tomato, along with those of human FTO, were subjected to analysis by employing the neighbor-joining method in MEGA7 software to construct a phylogenetic tree.

### m6A-seq and data analysis

Methylated RNA immunoprecipitation sequencing (MeRIP-seq) at Novogene (Beijing, China) was used to examine the RNA m6A modification. In summary, 2 μg of RNA was isolated from both the WT and *Slalkbh9b*. A simpliNano spectrophotometer (GE Healthcare) and an Agilent 2100 bioanalyzer (Agilent) were used to measure the concentration and integrity of extracted RNA, respectively. During the immunoprecipitation experiment, the fragmented RNA (about 100 nucleotides) underwent immunoreaction for 120 minutes at 4°C using a polyclonal anti-m6A antibody (Merk Millipore). The Ovation SoLo RNA-Seq System Core Kit (NuGEN) was then utilized to create the library using the immunoprecipitated RNA or input RNA. Following conventional procedures, the constructed sequencing libraries were subjected to high-throughput analysis employing either Illumina Novaseq or HiSeq systems, generating 150 bp paired-end reads.

The initial step involved processing the raw data (raw readings) in the fastq format using fastp (version 0.19.11). In this phase, clean reads have been obtained through the elimination of adapter-containing reads, poly-N reads, and reads of poor quality from the raw data. Simultaneously, calculations were made for the Q20, Q30, and GC content of the clean data. All further analyses were conducted using the high-quality data that was provided. After mapping the reads onto the standard genome, and the exomePeak R package (version 2.16.0) was utilized to identify the m6A peak in each anti-m6A immunoprecipitation category, by using the matching input samples acting as controls. For all data sets, a q-value of 0.05 was used to assess enrichment. HOMER (version 4.9.1) found each group’s m6A-enriched motifs. The peak-calling findings demonstrated every single peak corresponding to a gene and had been embedded in its exon. These genes were designated as peak-related genes. Subsequently, Subsequently, KOBAS software (version 3.0) was employed to examine the statistical enrichment of genes linked to peaks within KEGG pathways.

### Electrophoretic mobility shift assays (EMSA)

The identical technique used for SlACS3 and SlACS8 expression was used for SlALKBH9B-His protein. The protein was then purified using the kit (Beyotime Biotechnology, Jiangsu, China) in preparation for further research. The EMSA was carried out with a Chemiluminescent EMSA Kit also provided by Beyotime Biotechnology, located in Saibaisheng Company in China prepared the biotin-labeled probe. Supplementary Table S5 provides a comprehensive list of five pairs of oligonucleotide probes used in this study. The experimental procedure was based on a previously reported method [51, 78]. We made minor changes based on it. The isolated protein was treated with five pairs of labeled probes each. Following the process, the protein was transformed into the nylon membrane, and its creation was seen. We subsequently employ ImageJ to assess the binding intensity based on the depth of the binding probes.

### Translation efficiency assay

Merchante and colleagues presented a method to judge translation efficiency [79]. In short, 5 g of tomato flower pedicel abscission zone (AZ) was pulverized into a fine powder using liquid nitrogen. The remaining portion was suspended in 15 mL of polysome extraction buffer (200 mM Tris–HCl (pH 9.0), 35 mM MgCl_2_, 200 mM KCl, 25 mM EGTA, 1% Triton X-100 (v/v), 1% IGEPALCA-630 (v/v), 5 mM DTT, 1 mM PMSF, 50 μg·mL^−1^ Chloramphenicol, and 100 μg∙mL^−1^ Cycloheximide) with gentle shaking for 20 minutes at 4°C. Then, carefully transfer 12.5 mL of the supernatant over 13.5 mL of sucrose buffer (contents: 50 μg∙mL^−1^ of chloramphenicol, 50 μg∙mL^−1^ of cycloheximide, 400 mM Tris–HCl (pH 9.0), 35 mM MgCl₂, 5 mM EGTA, 200 mM KCl, 5 mM DTT, and 200 mM KCl. The complexes at the bottom were immersed again in 300 μL of DEPC-treated water after the supernatant was carefully removed following a 4-hour centrifugation at 20 0000 × g at 4°C. The plant RNA extraction kit (AG, Hunan, China) was utilized to isolate the polysomal and total RNAs, which were then used for the qRT-PCR study, as indicated earlier. The efficiency of translation was determined through comparing the mRNA abundance in polysomal RNA to that in total RNA, utilizing the CT 2^(−ΔCT)^ method [80]. The PCR amplification primers are listed in Supplementary Table S5.

### Statistical analysis

Three distinct biological replicates were used in each experiment. The chart or legend contains the specific statistical parameters for each experiment. Utilizing GraphPad Prism (v9), statistical analysis was carried out.

## Supplementary Material

Web_Material_uhaf173

## Data Availability

The National Center for Biotechnology Information has received the m6A-seq data gathered from this investigation. The paper and the Supplementary Information files provide all the data that backs up the study’s findings. Moreover, this manuscript encompasses source data.
