# Supplementary material for: SlALKBH9B is involved in drought-induced flower drop by regulating ethylene production
Source: Hortic Res. 2025 Jul 7;12(10):uhaf173. doi: 10.1093/hr/uhaf173 (PMC12528652; doi:10.1093/hr/uhaf173)
Supplement: Web_Material_uhaf173 [file web_material_uhaf173.zip › Figure S7.pdf]

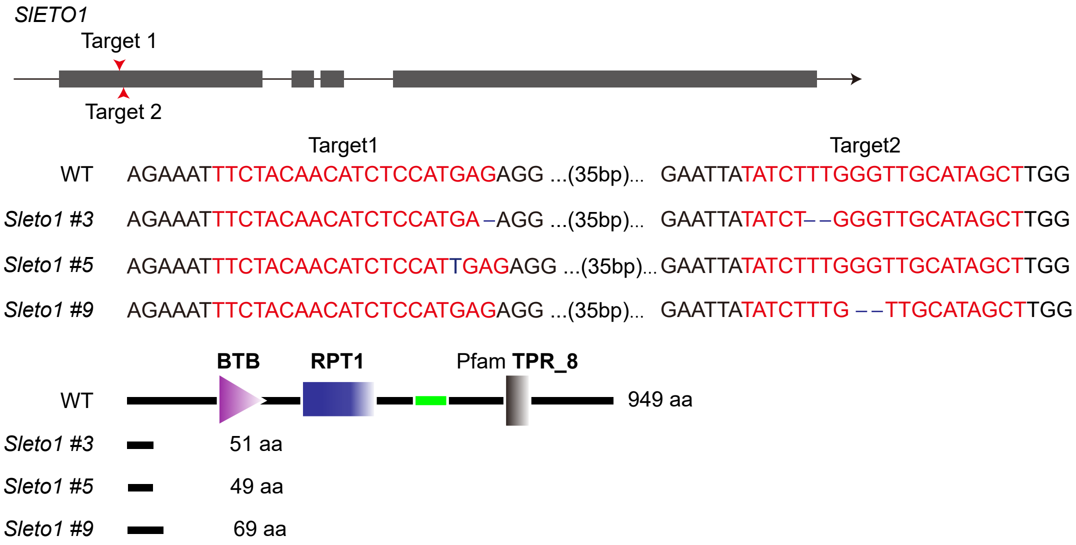
SlETO1
Target 1
Target 2
Target1
Target2
WT AGAAATTTCTACAACATCTCCATGAGAGG ...(35bp)... GAATTATATCTTTGGGTTGCATAGCTTGG
Sleto1 #3 AGAAATTTCTACAACATCTCCATGA–AGG ...(35bp)... GAATTATATCT– –GGGTTGCATAGCTTGG
Sleto1 #5 AGAAATTTCTACAACATCTCCATTGAGAGG ...(35bp)... GAATTATATCTTTGGGTTGCATAGCTTGG
Sleto1 #9 AGAAATTTCTACAACATCTCCATGAGAGG ...(35bp)... GAATTATATCTTTG – –TTGCATAGCTTGG
BTB
RPT1
Pfam TPR_8
WT
949 aa
Sleto1 #3
51 aa
Sleto1 #5
49 aa
Sleto1 #9
69 aa
