# Supplementary material for: SlALKBH9B is involved in drought-induced flower drop by regulating ethylene production
Source: Hortic Res. 2025 Jul 7;12(10):uhaf173. doi: 10.1093/hr/uhaf173 (PMC12528652; doi:10.1093/hr/uhaf173)
Supplement: Web_Material_uhaf173 [file web_material_uhaf173.zip › Supplemental Figure.pptx]

## Slide 1
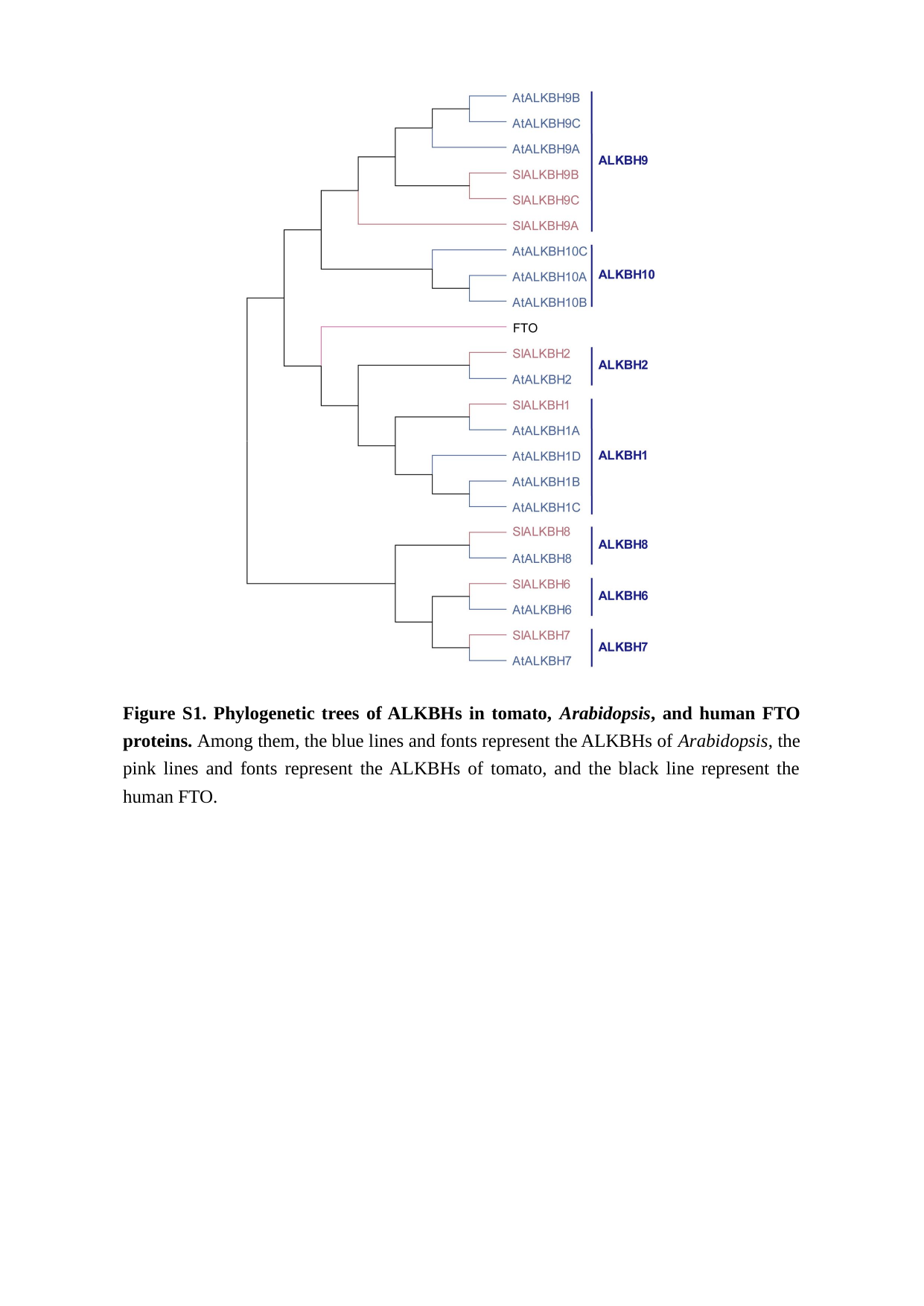

Figure S1. Phylogenetic trees of ALKBHs in tomato, Arabidopsis, and human FTO proteins. Among them, the blue lines and fonts represent the ALKBHs of Arabidopsis, the pink lines and fonts represent the ALKBHs of tomato, and the black line represent the human FTO.

## Slide 2
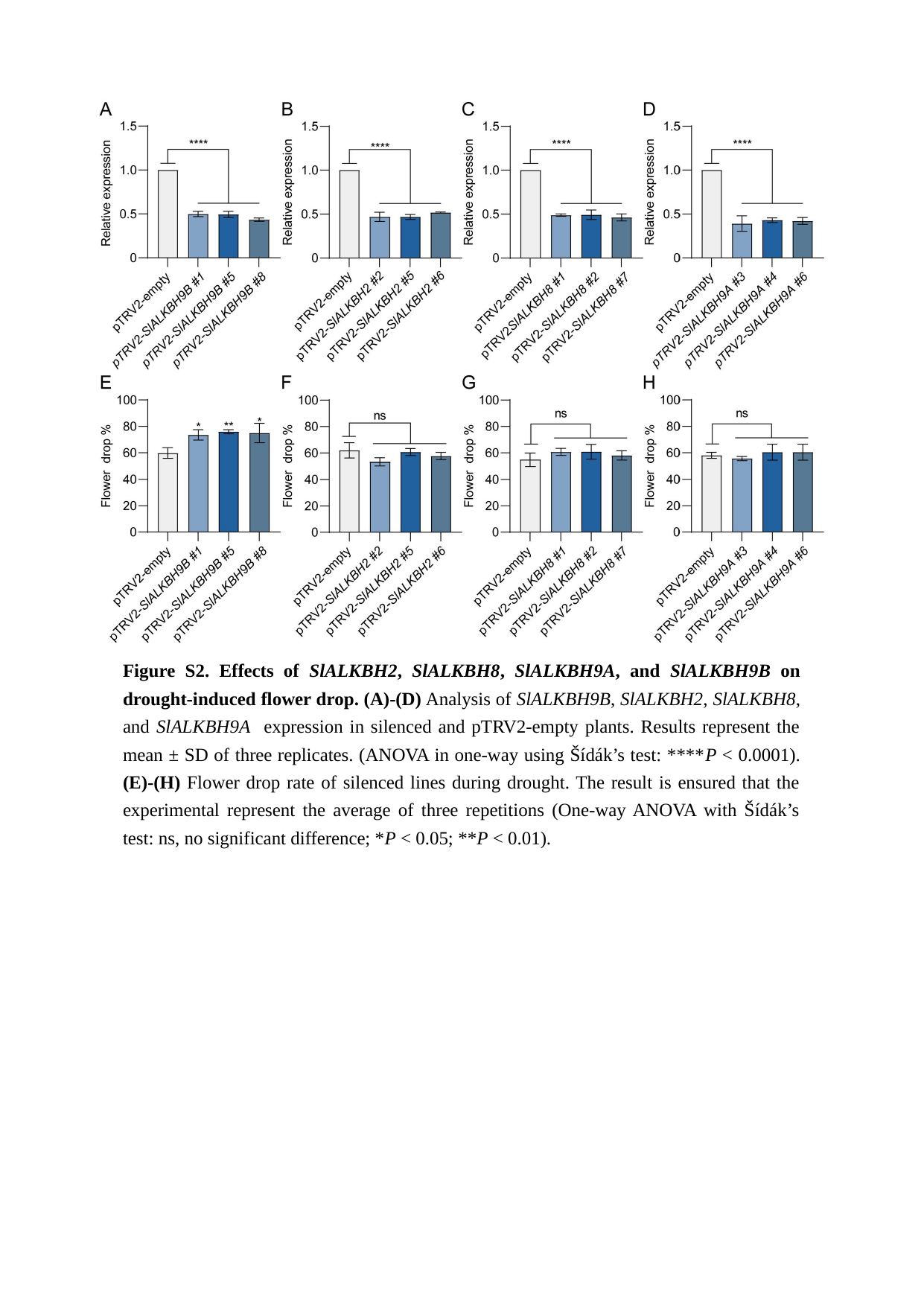

Figure S2. Effects of SlALKBH2, SlALKBH8, SlALKBH9A, and SlALKBH9B on drought-induced flower drop. (A)-(D) Analysis of SlALKBH9B, SlALKBH2, SlALKBH8, and SlALKBH9A expression in silenced and pTRV2-empty plants. Results represent the mean ± SD of three replicates. (ANOVA in one-way using Šídák’s test: ****P < 0.0001). (E)-(H) Flower drop rate of silenced lines during drought. The result is ensured that the experimental represent the average of three repetitions (One-way ANOVA with Šídák’s test: ns, no significant difference; *P < 0.05; **P < 0.01).

## Slide 3
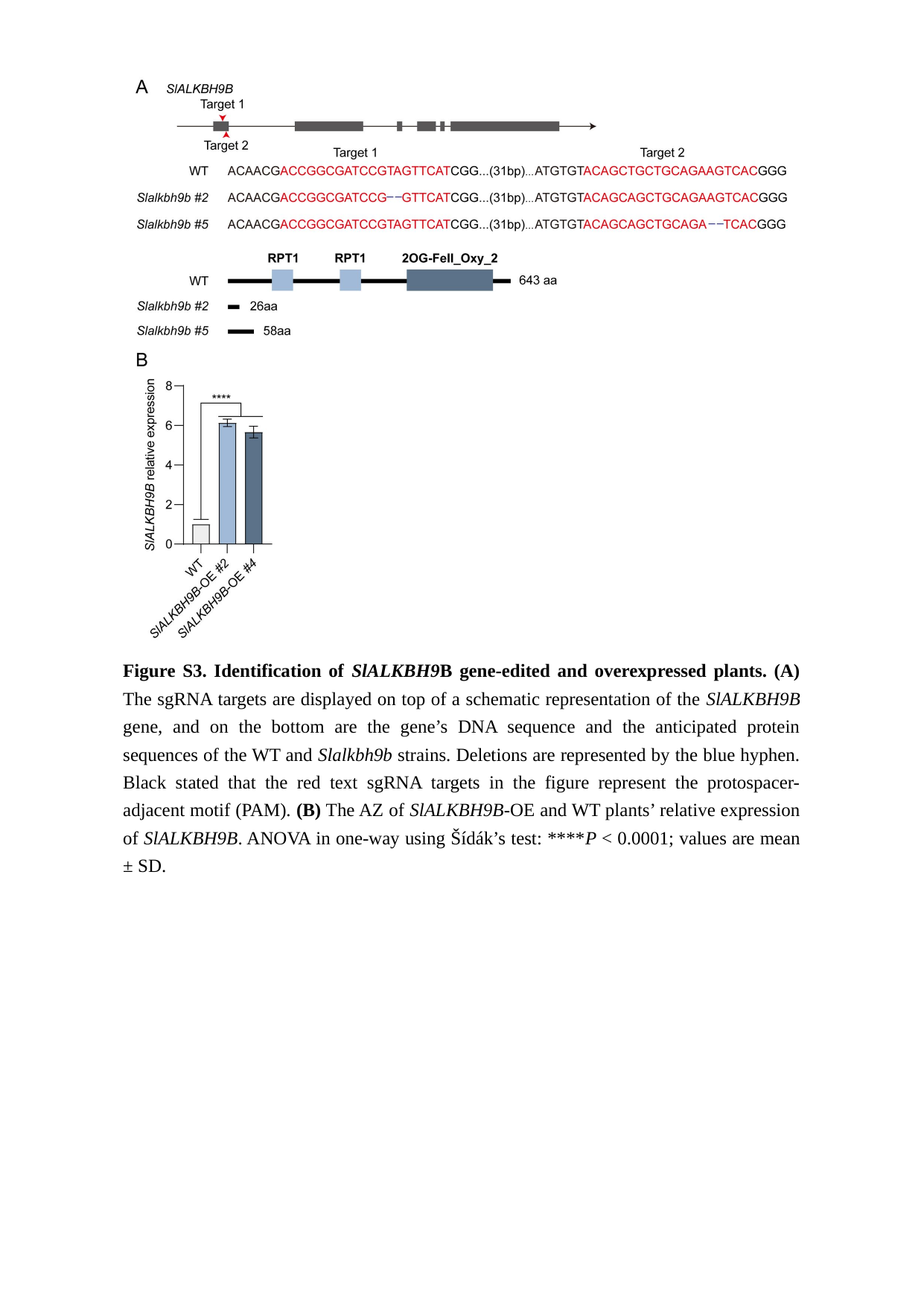

Figure S3. Identification of SlALKBH9B gene-edited and overexpressed plants. (A) The sgRNA targets are displayed on top of a schematic representation of the SlALKBH9B gene, and on the bottom are the gene’s DNA sequence and the anticipated protein sequences of the WT and Slalkbh9b strains. Deletions are represented by the blue hyphen. Black stated that the red text sgRNA targets in the figure represent the protospacer-adjacent motif (PAM). (B) The AZ of SlALKBH9B-OE and WT plants’ relative expression of SlALKBH9B. ANOVA in one-way using Šídák’s test: ****P < 0.0001; values are mean ± SD.

## Slide 4
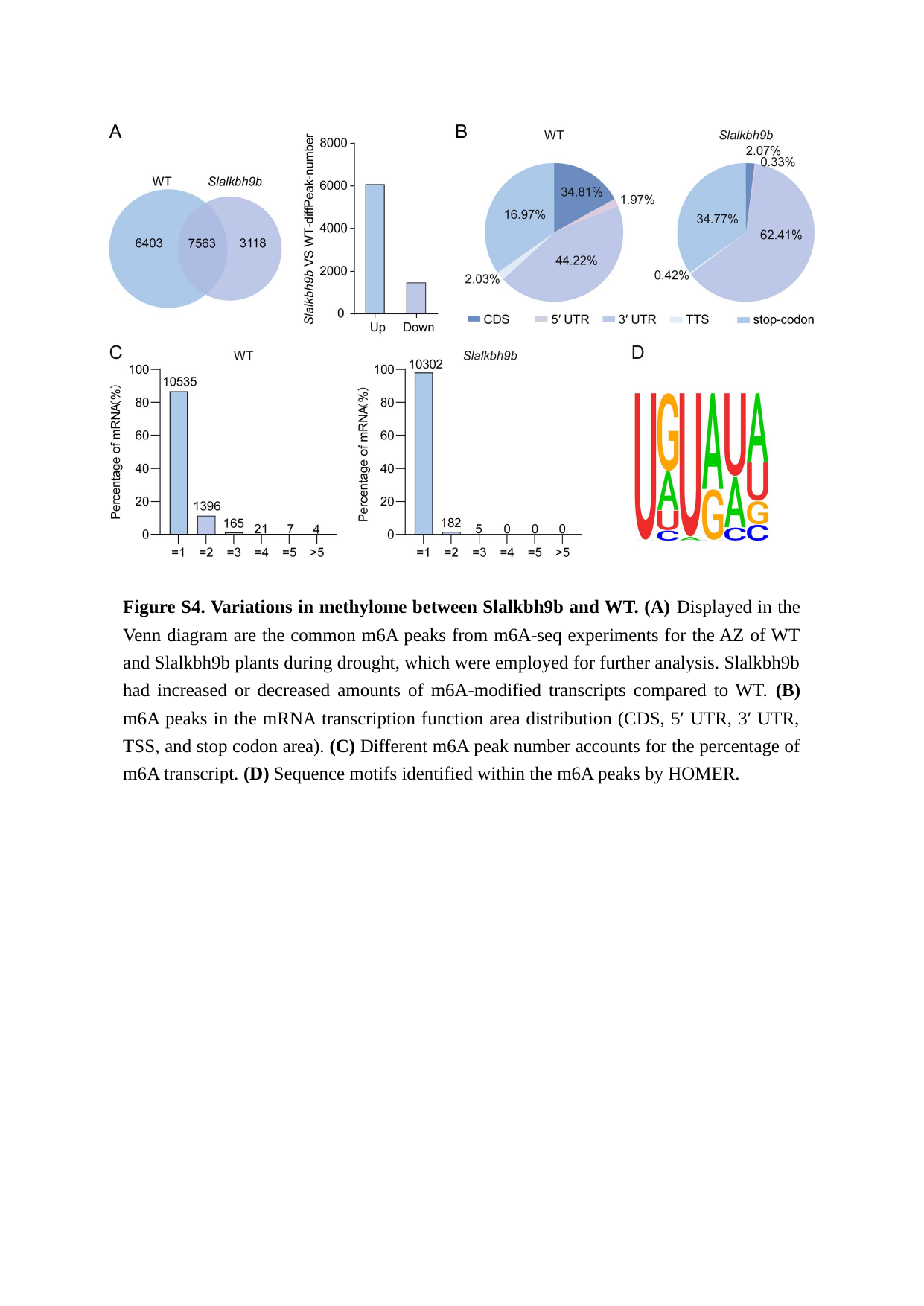

Figure S4. Variations in methylome between Slalkbh9b and WT. (A) Displayed in the Venn diagram are the common m6A peaks from m6A-seq experiments for the AZ of WT and Slalkbh9b plants during drought, which were employed for further analysis. Slalkbh9b had increased or decreased amounts of m6A-modified transcripts compared to WT. (B) m6A peaks in the mRNA transcription function area distribution (CDS, 5′ UTR, 3′ UTR, TSS, and stop codon area). (C) Different m6A peak number accounts for the percentage of m6A transcript. (D) Sequence motifs identified within the m6A peaks by HOMER.

## Slide 5
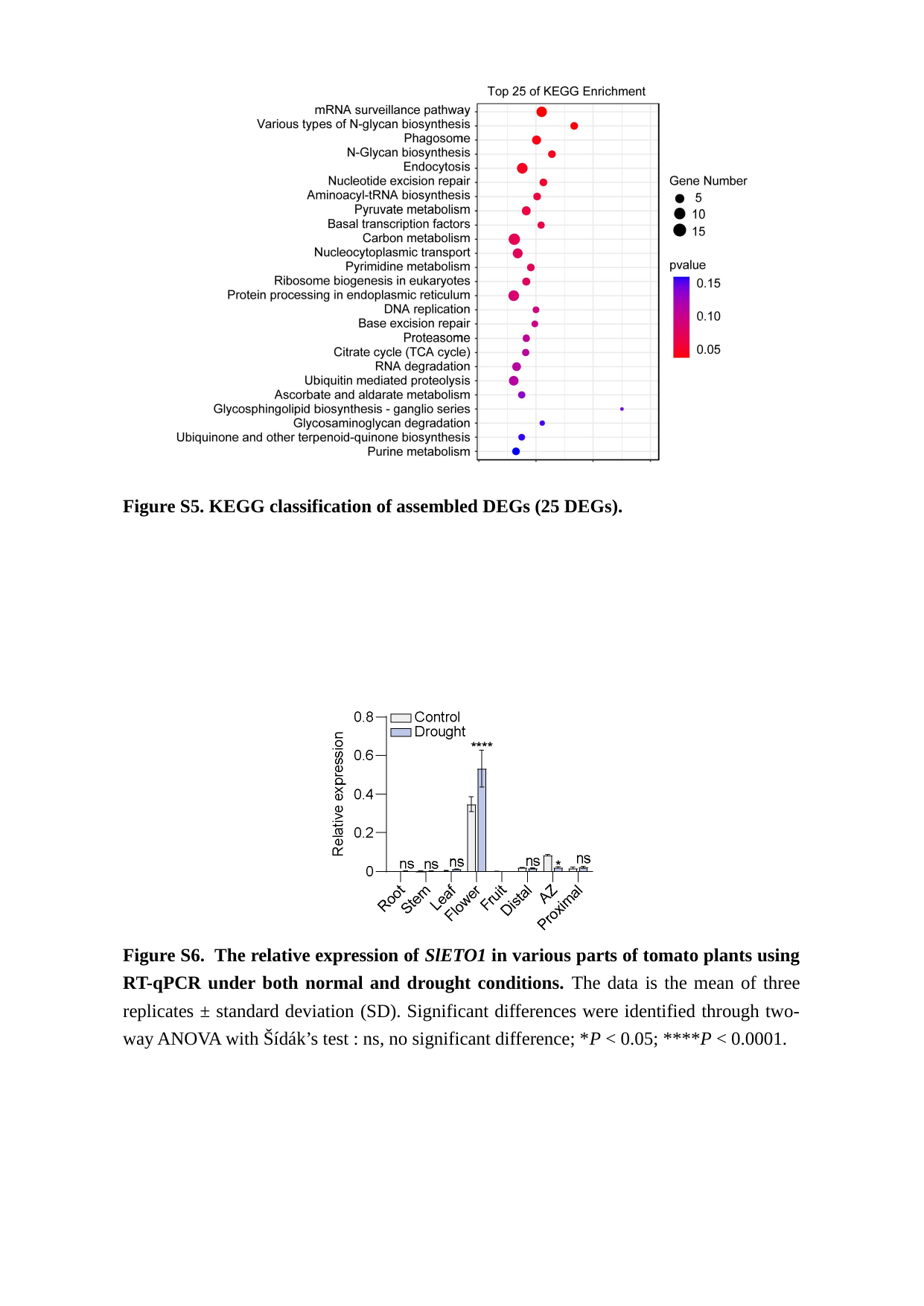

Figure S5. KEGG classification of assembled DEGs (25 DEGs).
Figure S6. The relative expression of SlETO1 in various parts of tomato plants using RT-qPCR under both normal and drought conditions. The data is the mean of three replicates ± standard deviation (SD). Significant differences were identified through two-way ANOVA with Šídák’s test : ns, no significant difference; *P < 0.05; ****P < 0.0001.

## Slide 6
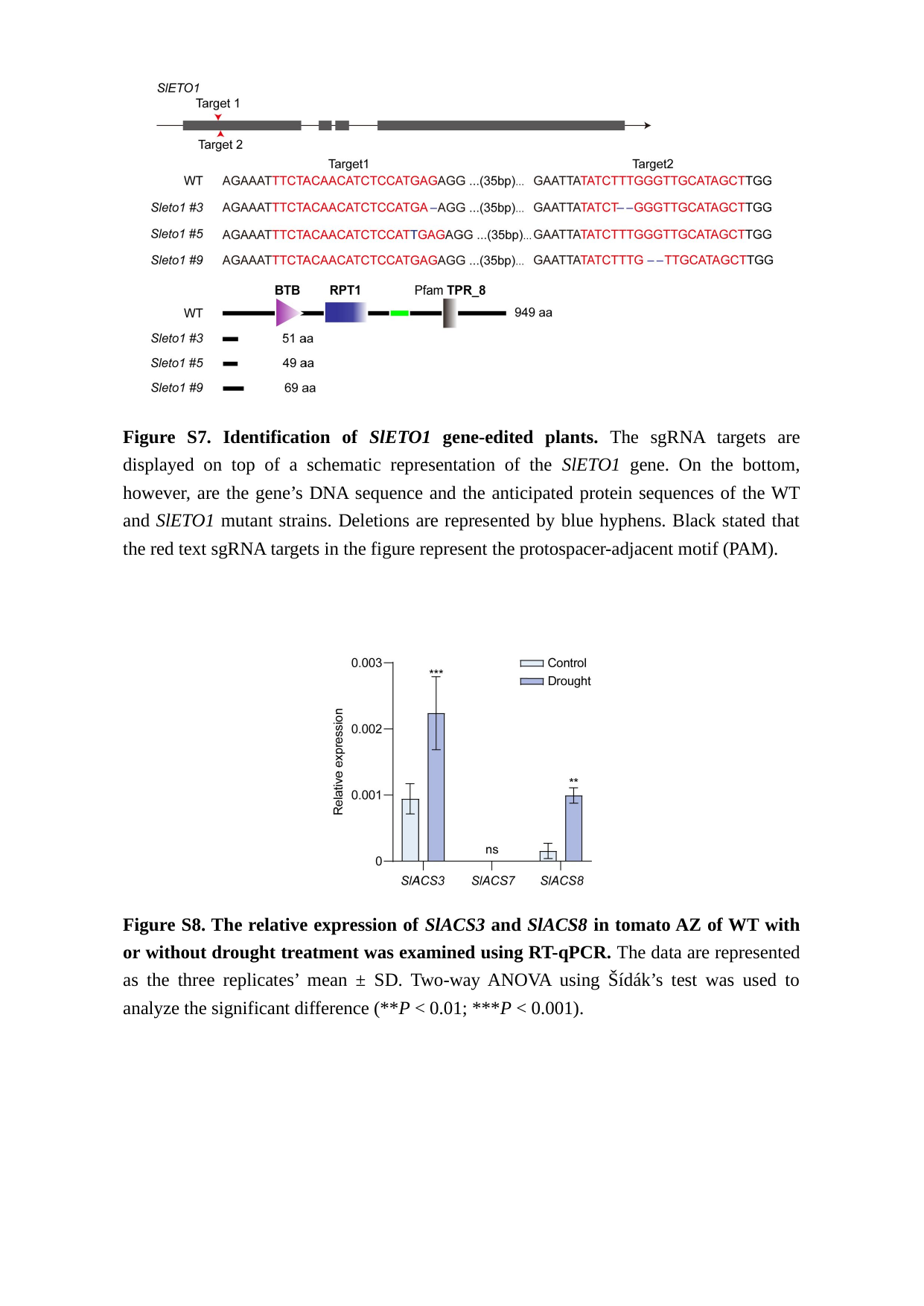

Figure S7. Identification of SlETO1 gene-edited plants. The sgRNA targets are displayed on top of a schematic representation of the SlETO1 gene. On the bottom, however, are the gene’s DNA sequence and the anticipated protein sequences of the WT and SlETO1 mutant strains. Deletions are represented by blue hyphens. Black stated that the red text sgRNA targets in the figure represent the protospacer-adjacent motif (PAM).
Figure S8. The relative expression of SlACS3 and SlACS8 in tomato AZ of WT with or without drought treatment was examined using RT-qPCR. The data are represented as the three replicates’ mean ± SD. Two-way ANOVA using Šídák’s test was used to analyze the significant difference (**P < 0.01; ***P < 0.001).

## Slide 7
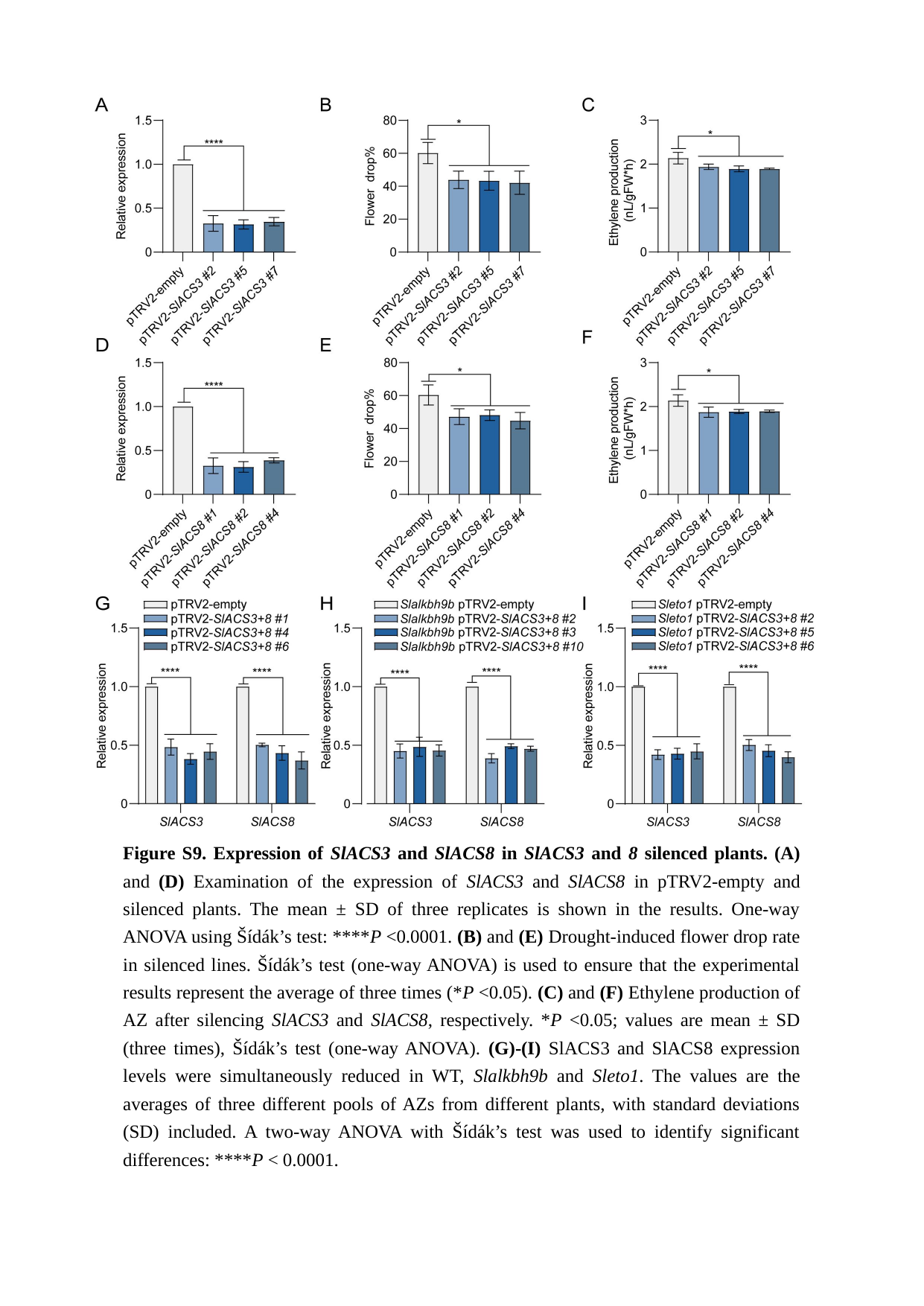

Figure S9. Expression of SlACS3 and SlACS8 in SlACS3 and 8 silenced plants. (A) and (D) Examination of the expression of SlACS3 and SlACS8 in pTRV2-empty and silenced plants. The mean ± SD of three replicates is shown in the results. One-way ANOVA using Šídák’s test: ****P <0.0001. (B) and (E) Drought-induced flower drop rate in silenced lines. Šídák’s test (one-way ANOVA) is used to ensure that the experimental results represent the average of three times (*P <0.05). (C) and (F) Ethylene production of AZ after silencing SlACS3 and SlACS8, respectively. *P <0.05; values are mean ± SD (three times), Šídák’s test (one-way ANOVA). (G)-(I) SlACS3 and SlACS8 expression levels were simultaneously reduced in WT, Slalkbh9b and Sleto1. The values are the averages of three different pools of AZs from different plants, with standard deviations (SD) included. A two-way ANOVA with Šídák’s test was used to identify significant differences: ****P < 0.0001.
