# Supplementary figures and images for: SlALKBH9B is involved in drought-induced flower drop by regulating ethylene production
Source: Hortic Res. 2025 Jul 7;12(10):uhaf173. doi: 10.1093/hr/uhaf173 (PMC12528652; doi:10.1093/hr/uhaf173)

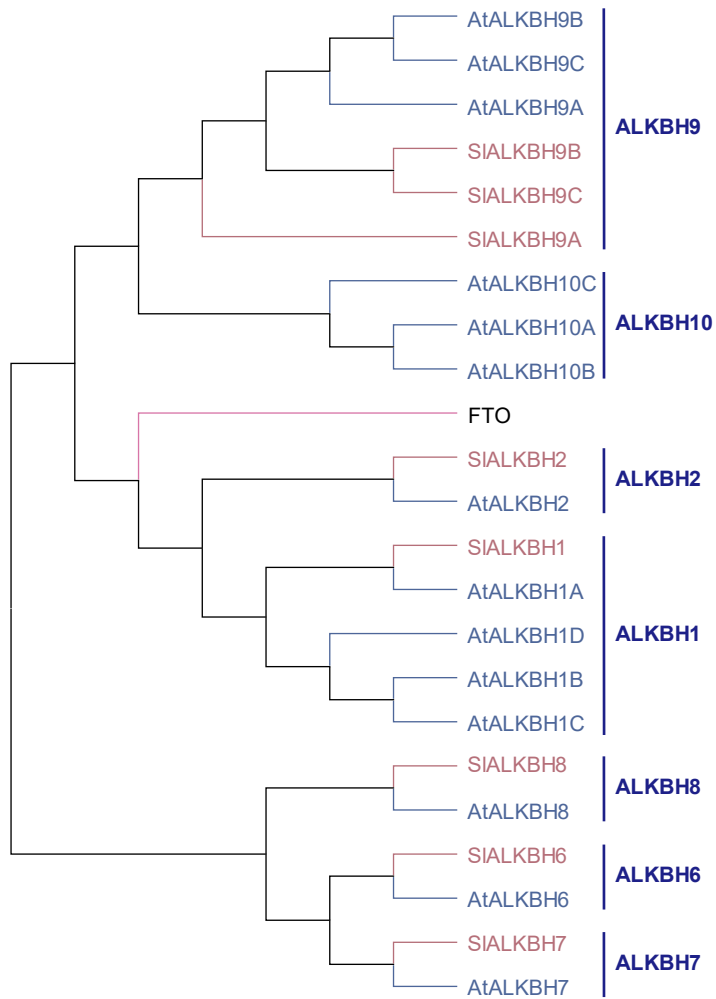

Supplement: Web_Material_uhaf173 [file web_material_uhaf173.zip › Figure S1.pdf]

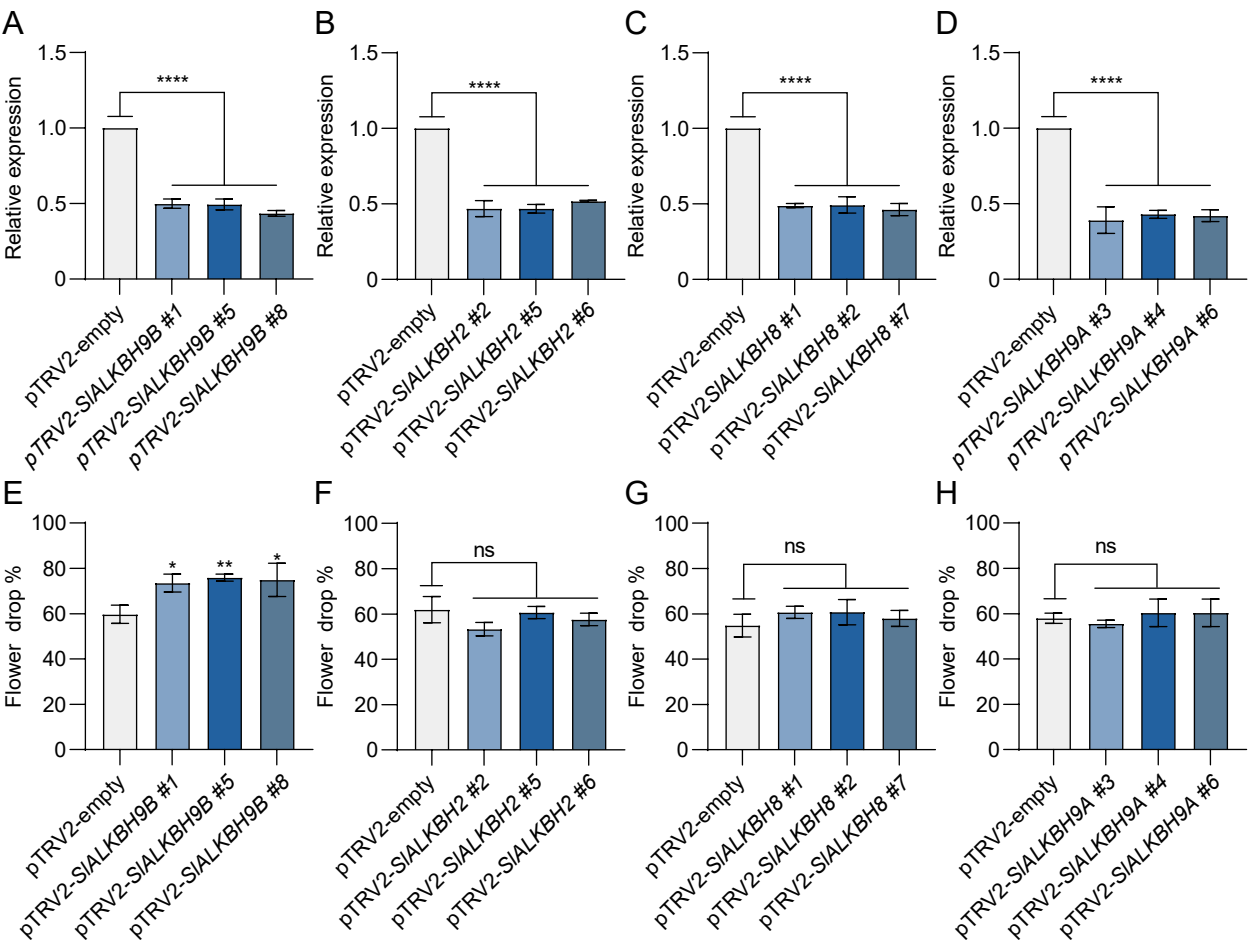

Supplement: Web_Material_uhaf173 [file web_material_uhaf173.zip › Figure S2.pdf]

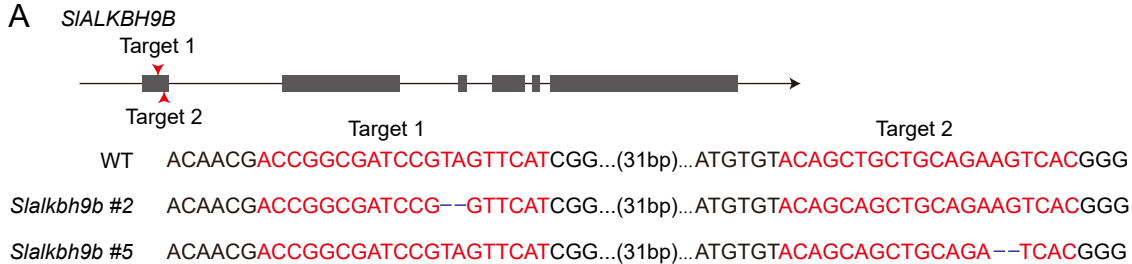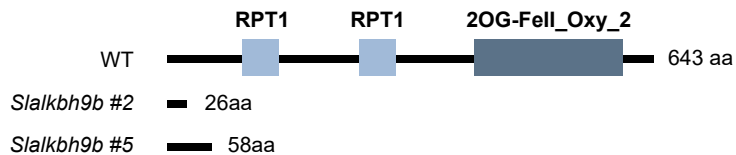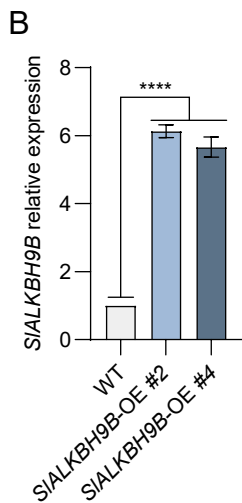

Supplement: Web_Material_uhaf173 [file web_material_uhaf173.zip › Figure S3.pdf]

**A**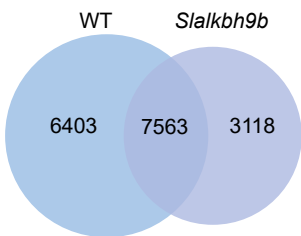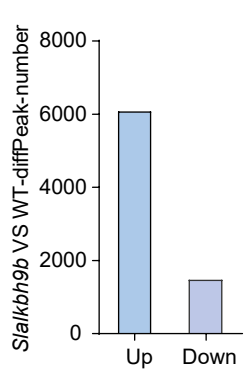**B**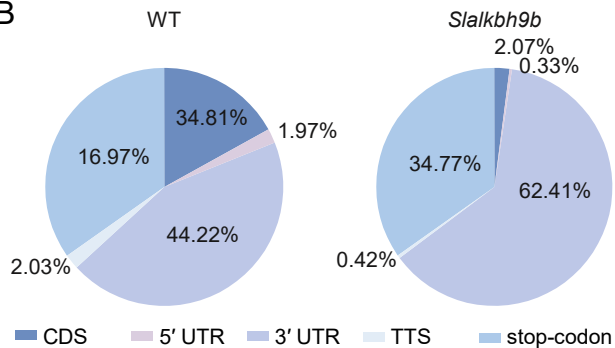**C**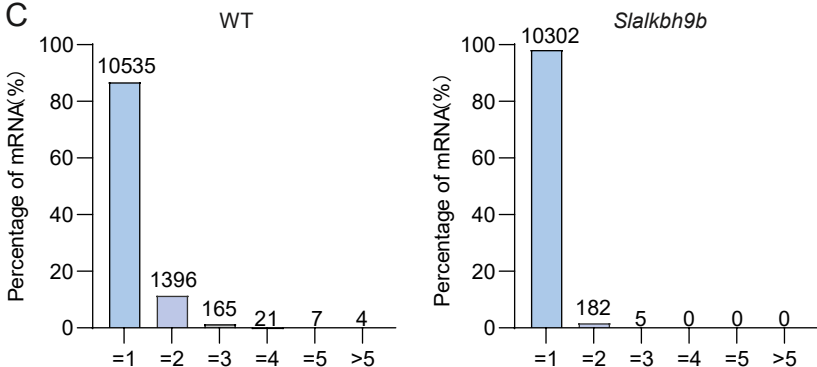**D**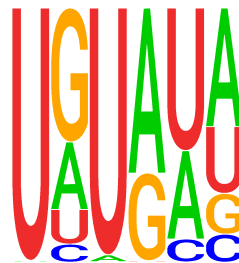

Supplement: Web_Material_uhaf173 [file web_material_uhaf173.zip › Figure S4.pdf]

Top 25 of KEGG Enrichment

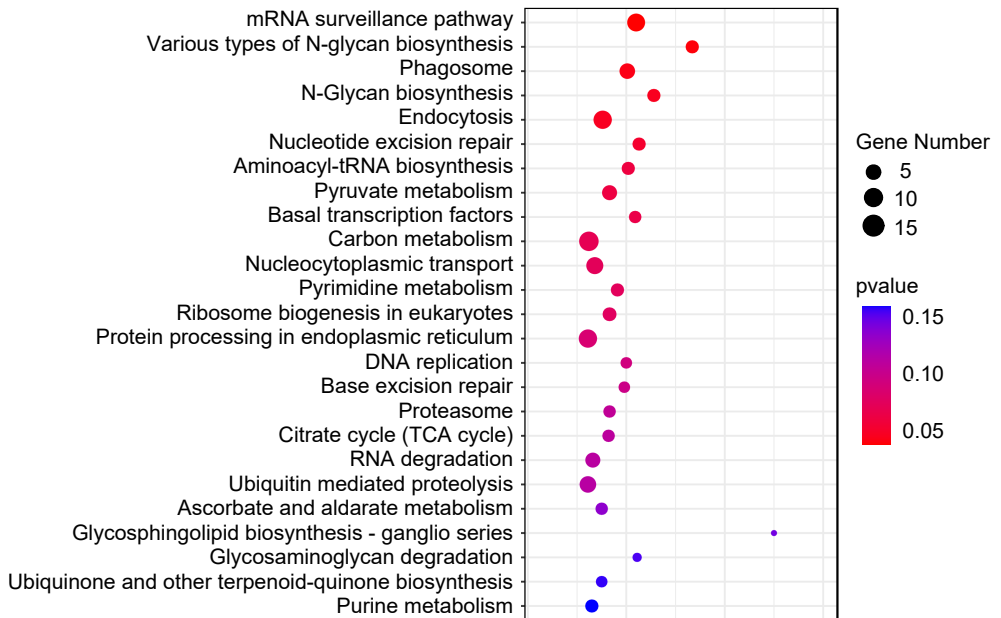

Supplement: Web_Material_uhaf173 [file web_material_uhaf173.zip › Figure S5.pdf]

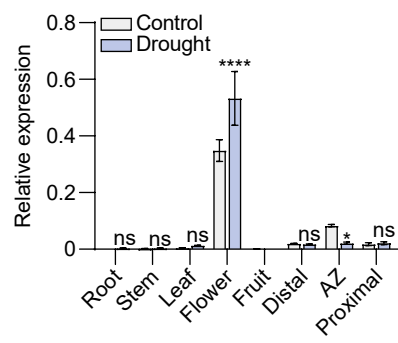

Supplement: Web_Material_uhaf173 [file web_material_uhaf173.zip › Figure S6.pdf]

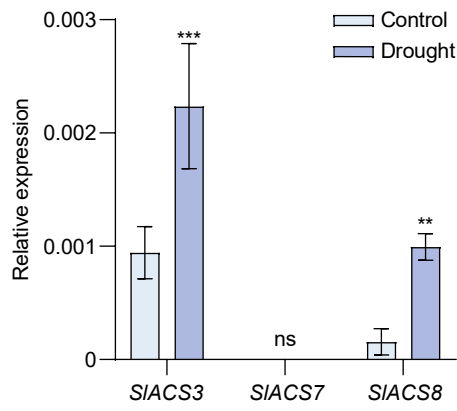

Supplement: Web_Material_uhaf173 [file web_material_uhaf173.zip › Figure S8.pdf]

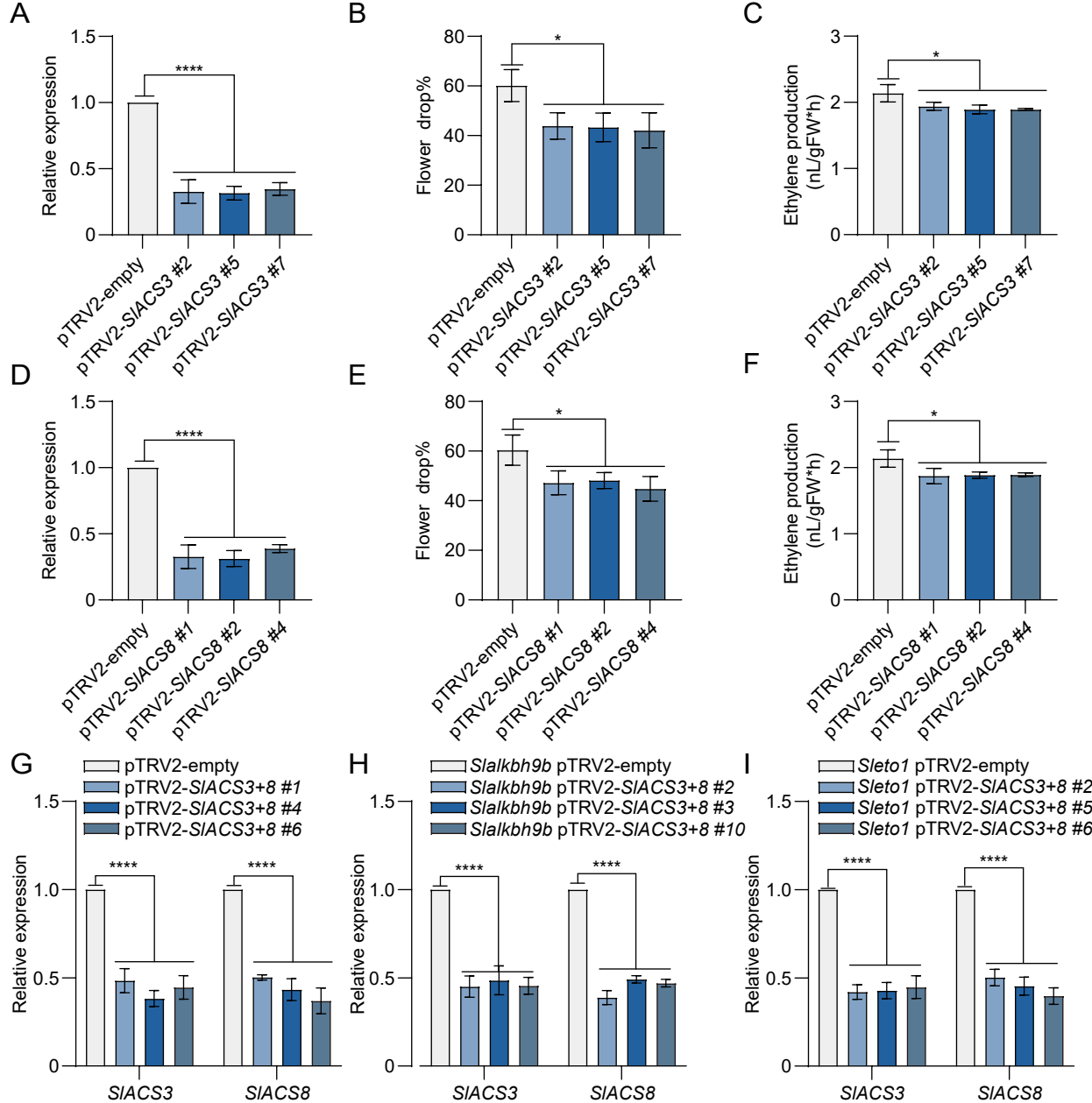

Supplement: Web_Material_uhaf173 [file web_material_uhaf173.zip › Figure S9.pdf]
